# Occult infection with hepatitis B virus PreS variants synergistically promotes hepatocellular carcinoma development in a high-fat diet context by generating abnormal ceramides

**DOI:** 10.1186/s12916-022-02481-3

**Published:** 2022-09-05

**Authors:** Chang Liu, Kun Chen, Fei Zhao, Lingling Xuan, Yuting Wang, Chungui Xu, Zhiyuan Wu, Dongmei Wang, Chunfeng Qu

**Affiliations:** State Key Lab of Molecular Oncology & Immunology Department, National Cancer Center/National Clinical Research Center for Cancer/Cancer Hospital, Chinese Academy of Medical Sciences and Peking Union Medical College, No 17 Panjiayuan South Lane, Chaoyang District, Beijing, 100021 People’s Republic of China

**Keywords:** Occult HBV infection, PreS mutations, High-fat diet, Liver inflammatory macrophages, Hepatocellular carcinoma

## Abstract

**Background:**

Some occult hepatitis B virus (HBV) infections are resulted from PreS mutations that reduce secretion of envelope protein (HBsAg). We investigated the ceramide amounts and species in hepatocytes infected with PreS variants that were isolated from HBsAg-seronegative patients with hepatocellular carcinoma (HCC) and the ceramide effects on autochthonous HCC development in murine models.

**Methods:**

HBV PreS/S regions from 35 HBsAg-seronegative HCC patients were sequenced. Hepatocyte cell lines and male C57BL/6J mouse livers were transfected with two PreS variant representatives. The ceramides with variated lengths of fatty acyl chains were quantified. Tumour development was examined in the HBV-transfected mice fed different diet types.

**Results:**

In HBsAg-seronegative HCC patients, nonneoplastic liver tissues harboured HBsAg and replication-competent HBV. The most frequently detected PreS/S variants carried mutations of altered amino acid properties in HBsAg compared with an isolate from one HBsAg-seronegative HCC patient. Hepatocyte infection with PreS variants caused HBsAg retention within the endoplasmic reticulum and generated more amounts of ceramides with C16:0 ceramide elevated the highest. Saturated fatty acids aggravated the PreS variant-infected hepatocytes to generate abnormal amounts and species of ceramides, which with HBV proteins synergistically activated NLRP3 inflammasome in liver inflammatory macrophages. Liver tumours were only detected in HBV-transfected mice fed high-fat diet, with higher tumour loads in the PreS variant-transfected, associated with abnormal ceramide generation.

**Conclusions:**

HBV PreS mutations which altered amino acid properties of envelope proteins inhibited HBsAg secretion. Hepatocyte infection with PreS variants generated abnormal ceramides which with HBV proteins coactivated NLRP3 inflammasome in liver macrophages to promote autochthonous HCC development.

**Supplementary Information:**

The online version contains supplementary material available at 10.1186/s12916-022-02481-3.

## Background

Hepatitis B virus (HBV) infection remains the leading cause of hepatocellular carcinoma (HCC) worldwide [1, 2]. Because of the hepatitis B vaccine and antiviral therapies, HCC major risk factors are changing. In the USA, individuals with metabolic disorders constitute the largest population attributable fraction (PAF) with HCC, and this fraction had increased from 25.8 to 36% between 2000 and 2011 [3]. The importance of metabolic (dysfunction) associated fatty liver disease (MAFLD) in HCC has thus received increasing attention [4]. To date, MAFLD prevalence in Asia, such as in China, where HCC is mainly due to HBV infection [1, 2], is gradually increasing. A study conducted between 2011 and 2012 revealed that MAFLD prevalence was 40.3% in Chinese adults 40 years old or older [5]. One population-based study reported that metabolic factors synergistically promoted HCC development in males with chronic HBV infection, particularly in patients with low levels of serum HBV-DNA copies [6]. However, another population-based study reported that metabolic syndromes were negatively associated with HCC in individuals with chronic HBV infection [7]. Hence, understanding the biological effects of metabolic disorders on HCC development is necessary for HBV-infected individuals to control their HCC development.

Clinically, occult HBV infection (OBI) has been frequently found in patients with chronic liver diseases and in certain asymptotic blood donors and individuals who have received the HBV vaccine. Currently, OBI is defined as the presence of replication-competent HBV (covalently closed circular DNA, cccDNA) in the liver and/or HBV-DNA in the blood of individuals who are seronegative for hepatitis B surface antigen (HBsAg) with currently available assays [8]. Hepatocyte endoplasmic reticulum (ER) is the key cellular organelle involved in HBV assembly and secretion. Both host immune responses and viral factors have certain effects on OBI. A subset of OBI people are associated to the infection with HBV genetic variants of PreS/S mutations [8, 9]. PreS/S encodes three different (large, middle and small) sized envelope proteins which are structurally related and play specific roles relating to protein transmembrane topology. In large envelop proteins, PreS domains, consisting of PreS1 and PreS2, project toward either the cytoplasm or the hepatocyte ER lumen [9]. Certain mutations, such as deletion or introduction of a stop codon in PreS1 or PreS2, cause an imbalance in the synthesis and secretion of the envelope protein, which results in HBsAg retention within hepatocyte ER, leading to ER stress and different liver diseases [8, 10]. We followed the cohort of individuals who received HBV vaccine at neonates [11] for 30 years and identified certain adults having replication-component HBV at serum levels of < 10,000 copies/ml with various PreS/S mutations [12, 13]. The outcome of OBI is largely unknown. However, a previous meta-analysis indicated that OBI was significantly associated with increased HCC risk, and the hepatocarcinogenesis is not clearly understood [8, 14].

Chronic liver inflammation in hepatocarcinogenesis has been highlighted [15, 16], though HBV integration is considered the most important driver [9, 16, 17]. Activation of NOD-like receptor protein 3 (NLRP3), which can be triggered by various endogenous “danger signals”, is a crucial step in the development of metabolism-related disorders and liver disease progression [18, 19]. One of the “danger signals” is the ceramide which exerts diverse effects depending on amide-linked fatty acid chain lengths varying from 14 carbons to 26 carbons (C14-C26) [18]. The de novo biosynthesis of ceramides begins with the condensation of serine and palmitoyl-CoA, which is catalysed by the serine palmitoyl-transferase enzyme. Myriocin, an enzyme inhibitor, can modulate ceramide de novo generation. The synthesis of ceramides with varying acyl chain lengths is regulated by six ceramide synthases (CerS1-CerS6) and dihydroceramide desaturase (DES1), which is encoded by the gene of *DEGS1.* The gene expression profiles of these synthases differ throughout the body. However, the liver is a key organ in lipid metabolism, and the ER is key to ceramide production. Given the importance of hepatocyte ER for HBV virion assembly/secretion and ceramide production, we therefore investigated ceramide species and concentrations in cells infected with PreS variants and ascertained their effects on HCC development in mice fed different diet types.

## Methods

### Human samples, cell lines and reagents

The protocol for using human samples was approved by the Ethics Committee of National Cancer Center/Cancer Hospital, Chinese Academy of Medical Sciences (NCC/CH, CAMS). Cell lines of HepG2 and Huh7 were purchased from ATCC, L02 from SIBS cell bank of Chinese Academy of Sciences. HBV envelop protein and HBV core protein were purchased from Jianan Biotechnology (Beijing, China). Palmitic acid (PA), fatty acid-free bovine serum albumin (FF-BSA), myriocin (Myr), C6-ceramide, thioglycolate and diethylnitrosamine (DEN) were purchased from Sigma-Aldrich (St. Louis, MO, USA). Detailed information for preparation of these reagents is provided in Additional file 1: Supplementary methods [15, 20–24].

### Cell transfection with HBV plasmids and treatment

For transfection of hepatocytes, the pcDNA3.1(-) was used to construct three different HBV plasmids. Ref-HBV contained 1.3 × HBV genome (nucleotides 1070-3215/1-1990) of an isolate (GenBank accession number GU434374) that was derived from one HBsAg-seropositive HCC patient. mtPreS1 contained 1.3 × HBV genome with mutated PreS1 at E54K-V90A-G102R, and mtPreS2 contained 1.3 × HBV genome with mutated PreS2 at V32L-I42T and 16-22 RVRGLYF deletion. Both mtPreS1 and mtPreS2 had identical sequences of S and other genes with Ref-HBV. Cells were cultured following the supplier’s instructions. For plasmid transfection, 5 × 10^5^/well of HepG2 cells in 6-well plates or 8 × 10^4^/well in 8-well chamber slides were cultured overnight and transfected with 2.5 μg or 300 ng plasmids by using Lipofectamine (Invitrogen, Carlsbad, CA, USA). In some experiments, the transfected cells were replaced with fresh medium 48 h later that contained different concentrations of PA or Myr to continue culture for variated times. The control medium contained FF-BSA, NaOH and DMSO which were used for dissolving PA or Myr respectively.

### Mice, diets and murine autochthonous HCC induction

C57BL/6J and *Nlrp3*^*−/−*^ (B6.129-*Nlrp3*^*tm1Hhf*^/J) mice were purchased from the Jackson Laboratory. High-fat (HF, D12492) and normal-chow (NC, D12450B) diet food was from Research Diets, USA. Study protocols involving mice were approved by the Institutional Animal Care and Use Committee at NCC/CH, CAMS. For inducing murine autochthonous HCCs, male C57BL/6J mice at 2 weeks old were intraperitoneally injected with 25 mg/kg body weight of DEN as previous reports [15, 24]. In some experiments, the 6-week-old mice received 6 μg HBV plasmids that were constructed using AAV-MCS vector, via tail vein hydrodynamic injection [25]. In some experiments, recipient mice received 5 × 10^6^ of bone marrow cells from donor mice via tail vein injection [15].

### HBV detection and analysis

Following our previous reports, we sequenced and analysed the HBV PreS/S regions [13] with the primers provided in Additional file 1: Table S1, examined the 3D structure of envelop protein using RaptorX structure prediction server [26] and determined HBV relaxed circular DNA (rcDNA) in the sera of HBsAg-seronegative HCC patients [12]. We detected HBV cccDNA in tissues using specific pan-genotypic primers following the protocol reported by Caviglia [27]. HBsAg secretion was measured using the quantification kit of Kehua Biotechnology (Shanghai, China). To determine HBsAg within ER, hepatocyte ER was isolated from total cellular proteins with an extraction kit (Baiaolaibo, Beijing, China) by density gradient ultracentrifugation based on the report [28]. HBsAg was released from ER by addition of RIPA lysis buffer.

### Ceramide measurement

High-performance liquid chromatography coupled to tandem mass spectrometry (HPLC-MS/MS) on an Agilent 6410B Triple Quad mass spectrometer (Santa Clara, CA, USA) was used to quantify the ceramide amounts and ceramide species in the State Key Laboratory of Bioactive Substance and Function of Natural Medicines at Institute of Materia Medica of CAMS. Briefly, the samples were prepared with three-phase solvent system, and chromatographic separation was conducted on a Spectra C8SR Column of 3-μm particle size (Peeke Scientific, CA, USA). Ceramide concentrations were quantified by Cer 18:0 standard curve with a linear concentration range of 0.2–200 ng/ml. Cer 17:0 was used as internal standards that were purchased from Avanti Polar Lipids (Alabaster, AL, USA). The numbers of quality control (QC) samples were more than 5% of the total analysed samples. The relative standard deviation of peak areas of sphingolipid metabolites between QC samples in each level was less than 30%. Detailed procedures for precision and accuracy of the measurement were described by Qu in the literature [29].

### Macrophage isolation

Peritoneal macrophages were prepared following the previously reported protocol [23]. Liver macrophages were prepared according to the literature [30]. Briefly, the intrahepatic infiltrated cells were separated from digested liver tissues and then applied on the 25%/50% Percoll gradient. After centrifugation at 800 *g* for 20 min, the cells at interface of the two-density cushion were collected.

### Immunohistochemistry (IHC) and immunofluorescent microscopy

Additional file 1: Table S2 provides the information of ELISA kits and antibodies used in the assays of immunohistochemistry, immunofluorescent, immunoblotting and flow cytometry. HBsAg in liver tissues was stained with IHC in paraffin-embedded human HCC samples with a mouse monoclonal antibody against S protein (H2F4, Bioss, Beijing, China). Co-localization of HBsAg with ER was examined using goat anti-GRP78 or rabbit anti-calnexin, with mitochondria using Mito-Tracker Red CMXRos after HBsAg staining. Images were acquired with a Nikon confocal microscope and analysed with Volocity software (PerkinElmer, Waltham, MA, USA). To analyse the infiltration of immune cells in mouse livers, paraffin-embedded sections were stained with rabbit anti-mouse CD45 polyclonal antibody, following addition of HRP-labelled goat anti-rabbit IgG. Sections were scanned using Aperio ScanScope software (Aperio Technologies). The infiltration of CD45-positive cells was analysed using Image-Pro Plus 6.0 (Media Cybemetics, USA).

### Immunoblotting, cytokine quantification and flow cytometry (FCM)

The assays were performed with standard laboratory protocols. For immunoblot analysis, 40 μg of proteins in each lane was loaded with β-actin as the loading control. The images were captured using Amersham Imager 600 (GE Healthcare, MA, USA), and the relative quantity of each band was analysed with ImageJ software (NIH, MD, USA). Commercialized ELISA kits were used to quantify the cytokine levels according to the manufacturer’s instructions. For FCM analysis, intrahepatic infiltrated cells were prepared as we previously reported [24] and incubated with PE/Cy7-conjugated anti-mouse CD45, PE-conjugated anti-mouse CD11b and PerCP/Cy5.5-conjugated anti-mouse F4/80. Data were acquired in LSR-II (BD, CA, USA) and analysed using Flowjo software (Tree Star, OR, USA).

### Quantitative real-time PCR (qRT-PCR)

Total mRNA from cells or liver tissues were isolated using TRIzol (Thermo Fisher Scientific. MA, USA) following the manufacturer’s protocol. The cDNA was synthesized using PrimeScript RT Reagents (Takara, Dalian, China). The gene transcription level was determined by qRT-PCR using SYBR Green reagent in a 7500 Fast Real-Time PCR system (Life Technology, CA, USA). The primer sequences are provided in Additional file 1: Table S3.

### Statistical analysis

GraphPad Prism 8 software was used for statistical analyses. The variables were firstly examined with the Shapiro-Wilk normality test. We used paired two-tailed Student’s *t-*test to compare the differences of two groups and one-way ANOVA to compare more than two groups. The difference of nonparametric data was compared with the Mann-Whitney *U* test. *P-*value of less than 0.05 was considered to be statistically significant.

## Results

### Nonneoplastic liver tissues in HBsAg-seronegative HCC patients harboured HBV cccDNA with extensive mutations in PreS/S regions

**Fig. 1:**
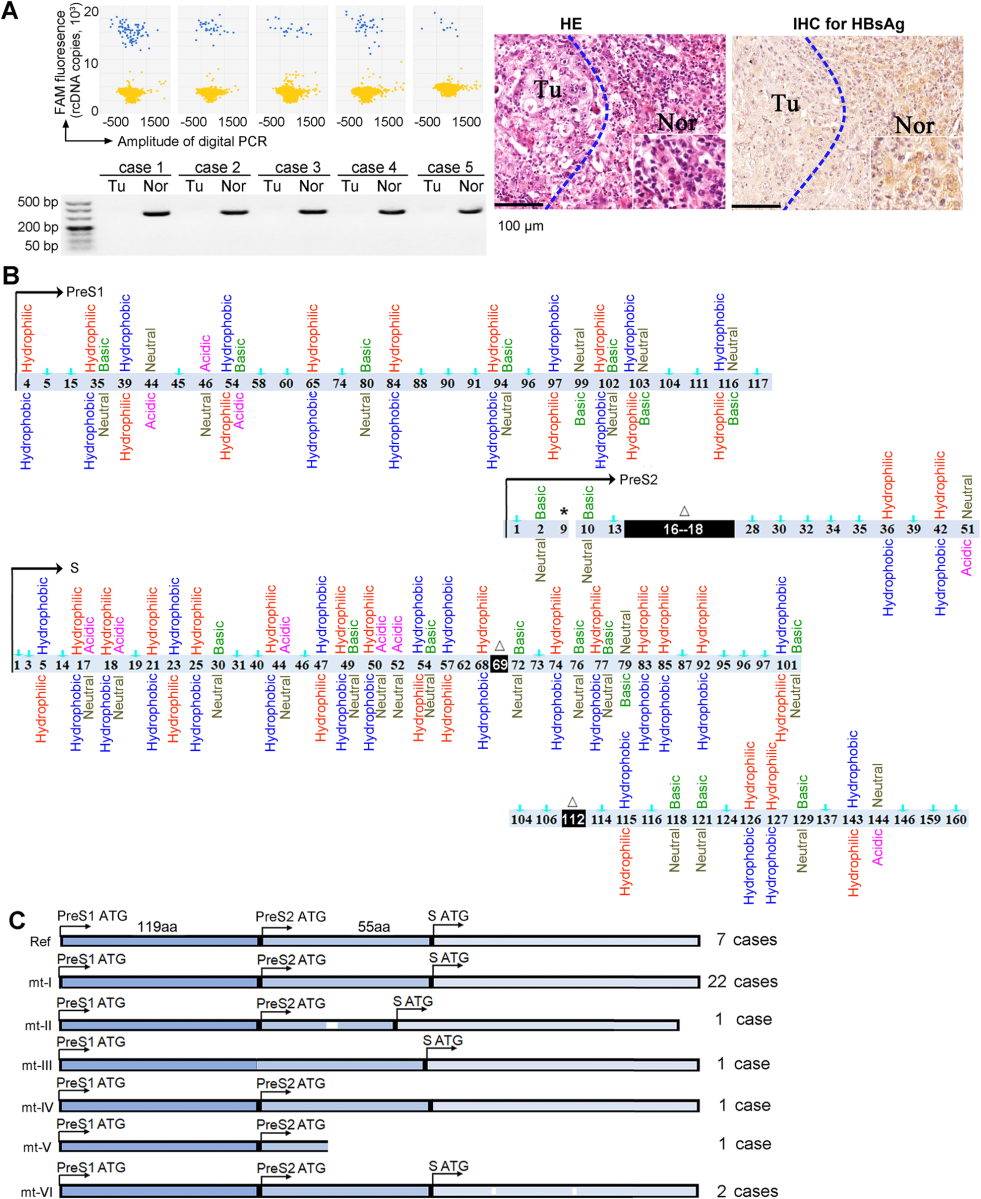
Replication-component HBV with extensive PreS/S mutations in in HBsAg-seronegative HCC patients. **A** Scatter diagrams show HBV rcDNA detected in 5 representative sera from the 35 HBsAg-seronegative HCC patients; the electrophoresis image shows the 332-bp products of the HBV gap region amplified from genomic DNA of liver tissues. Histological images show the representative HBsAg immunohistochemistry staining of tissue sections from the 35 HCC patients. **B** Mutations across PreS/S regions in 35 HBsAg-seronegative HCC patients. The isolate GU434374 from one HBsAg-seropositive HCC patient was used as the reference (Ref-HBV). Digits indicate the amino acid (AA) sites where mutations occurred. AA properties of Ref-HBV are shown below the digits, the mutated are above digits. Arrows indicate mutations occurred with no alteration in AA properties. “△” indicates deletion, “*” indicates stop codon. **C** PreS/S AA sequences in 7 HBsAg-seronegative HCC patients were identical to Ref-HBV. Six types of PreS/S mutations were recognized in the other 28 patients. mt-I (22 cases), PreS/S AA properties differ from Ref-HBV; mt-II (1 case), PreS2 deletion at 16–22 and different AA properties from Ref-HBV; mt-III (1 case), a mutated PreS2 start codon; mt-IV (1 case), a mutated S start codon; mt-V (1 case), a new stop codon introduced at position 9 of PreS2; mt-VI (2 cases), deletions at position 69 and 112 of S gene

We recognized 236 HBsAg-seronegative HCC patients after reviewing 1567 cases of histology-confirmed HCCs that had been diagnosed in the China NCC [31] (Additional file 1: Table S4). These HBsAg-seronegative HCC patients presented with HBV-DNA in serum at levels < 1000 IU/ml. We then sampled 35 cases to confirm the presence of replication-competent HBV in these HBsAg-seronegative HCC patients. HBV rcDNA was detectable at a concentration of 10^2^–10^3^ copies/ml in sera of the 35 patients. HBV cccDNA was detected in all (100%) of the nonneoplastic liver tissues, but in 6 (17%) of the paired tumour tissues. An IHC analysis showed that HBsAg was detectable in all 35 nonneoplastic liver tissues and in some HCC tissues (Fig. 1A).

We sequenced the HBV PreS/S regions that were obtained from the blood and from liver tissues, The sequences were deposited in GenBank with accession numbers of MW422170: MW422204. An alignment analysis showed that the amino acid (AA) sequences in PreS/S regions from blood and from liver tissue of each individual were identical (Additional file 1: Fig. S1). Phylogenetic analysis showed that all the isolates were HBV genotype C2. However, the PreS/S AA sequences differed between patients (Additional file 1: Fig. S2). We then compared the PreS/S AA properties of the isolates derived from the 35 HBsAg-seronegative HCC patients with the Ref-HBV, which was obtained from one HBsAg-seropositive HCC patient. The 35 isolates harboured different variations in PreS/S regions, including in-frame deletion or stop codon mutations as previously reported [10, 32]. However, most mutations altered the AA properties (Fig. 1B). Six variant types of PreS/S mutations were identified from 28 patients. The most frequently detected variants harboured mutations with altered AA properties leading to the conversion of hydrophobic properties into hydrophilic properties, and vice versa, and charge modifications (Fig. 1C).

### Infection with PreS variants that altered AA properties inhibited HBsAg secretion

**Fig. 2:**
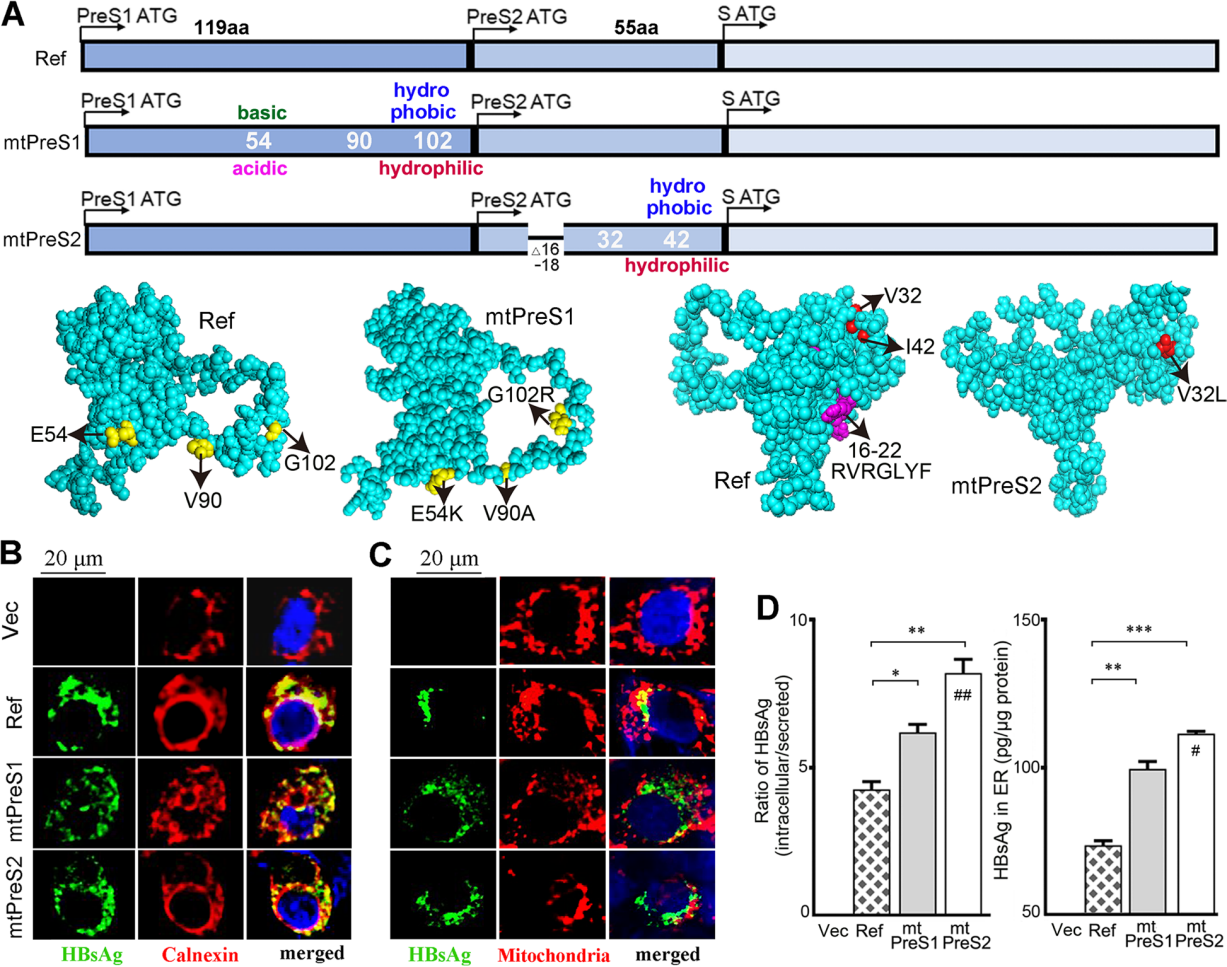
Infection with PreS variants that altered AA properties led to envelop protein retention within hepatocytes. **A** Schematic PreS/S regions of Ref-HBV, mtPreS1 and mtPreS2 based on the isolates obtained from 35 HBsAg-seronegative HCC patients. Shown are the predicted 3D structures of large envelop proteins encoded by Ref-HBV, mtPreS1 and mtPreS2 respectively using the RaptorX structure prediction sever (http://raptorx.uchicago.edu/). **B**, **C** In 8-well chamber slides, 8 × 10^4^/well of HepG2 cells were transfected with 300 ng of Ref-HBV, mtPreS1 or mtPreS2 plasmids respectively. Forty-eight hours later, the cells were double-stained **B** with antibodies against HBsAg (green) and calnexin (red, ER marker) or double-stained **C** against HBsAg and mitochondria (red) with Mito-Tracker Red CMXRos. Representative images are one of three independent experiments. DAPI (blue)-stained nuclei. **D** In 6-well plates, 5 × 10^5^/well of HepG2 cells were transfected with 2.5 μg of different HBV plasmids, respectively, and cultured for 48 h. The supernatant was collected, and cells were washed twice with PBS and harvested. Hepatocyte ER was further separated by density gradient ultracentrifugation from total cellular proteins. HBsAg in supernatant (secreted) and in total cell lysates (intracellular) were quantified using an ELISA kit. Bar graphs (mean ± SEM) show the ratio of intracellular to secreted HBsAg after normalizing to total cellular proteins (left), and the amount of HBsAg in ER organelle (right), from three independent experiments. Each group was triplicated in one experiment. The same amount of pcDNA3.1 (Vec) was used as transfection control. The map of different plasmids was provided in Additional file 1: Fig. S3. Differences of the HBsAg secretion in differently transfected cells were compared with one-way ANOVA analysis. *P < 0.05, **P < 0.01, ***P < 0.001 between Ref-HBV and mtPreS1 or mtPreS2; ^#^*P <* 0.05, ^##^*P <* 0.01 between mtPreS1 and mtPreS2

HBV envelope proteins perform specific roles relating to the protein transmembrane topology [8, 9]. Because many HBsAg-seronegative HCC patients were infected with HBV variants that altered the PreS AA properties (Fig. 1), we hypothesized that HBV large envelope proteins might change the transmembrane topology that leads to HBsAg retention within hepatocyte ER. Two HBsAg-seronegative HCC patients carried PreS1 E54K, V90A and G102R mutations (named mtPreS1), and one patient carried PreS2 mutations of V32L, I42T, 16-22 (RVRGLYF) deletion (named mtPreS2). We analysed the 3D structure of the large envelope protein using the RaptorX structure prediction server. The structure of the envelope protein encoded by mtPreS1 or mtPreS2 was profoundly different from the structure of the envelope protein encoded by Ref-HBV (Fig. 2A).

To confirm this finding, we constructed the plasmids of mtPreS1 and mtPreS2 based on the Ref-HBV genome. Plasmid of mtPreS1 was mutated only in the PreS1 sequence, and mtPreS2 was mutated only in the PreS2, and both plasmids shared identical S sequences and other sequences with Ref-HBV plasmid (Additional file 1: Fig. S3). Hepatocyte cell lines were transfected with these plasmids to mimic HBV infection. A confocal microscope analysis on the transfected HepG2 cells showed that HBsAg mainly localized to the ER which was positive for calnexin (Fig. 2B) and GRP78 (Additional file 1: Fig. S4). Some HBsAg also localized to the mitochondria (Fig. 2C). We collected the culture medium and harvested transfected cells respectively. ER compartment was separated from the cells by density gradient ultracentrifugation of total cellular protein. HBsAg amount in the supernatant, in the total cells and in the ER was determined using quantitative ELISA. The transfected HepG2 cells generated similar amounts of envelope proteins after transfection with Ref-HBV, or mtPreS1, or mtPreS2. However, more HBsAg was found within cells and in the ER compartment after HepG2 transfected with mtPreS1 or mtPreS2 than in those transfected with Ref-HBV (Fig. 2D). We transfected L02 and Huh7 cells and observed the same results as those obtained in HepG2 cells (Additional file 1: Fig. S5).

### Transfection of HBV PreS variants augmented hepatocyte generation of abnormal ceramides

**Fig. 3:**
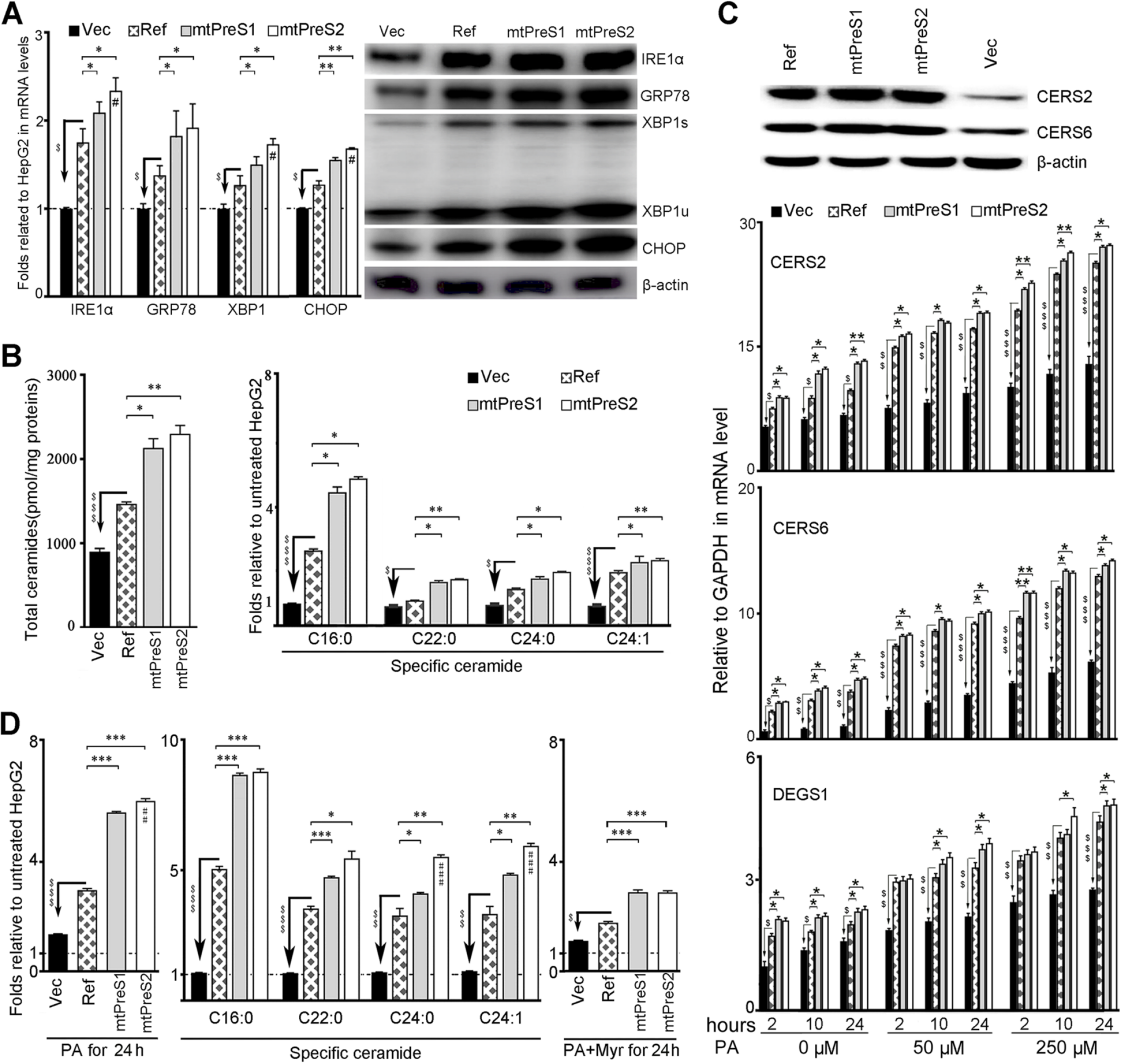
Infections with PreS variants induced hepatocyte ER stress and augmented the generation of abnormal ceramides. HepG2 cells were transfected respectively with different HBV plasmids as described in Fig. 2D and cultured for 48 h. Empty-vector (Vec) was the transfection control. **A** Transcriptional levels (mean ± SEM) of the specified genes from three independent experiments determined by qRT-PCR. The other genes are provided in Additional file 1: Fig. S6. Image shows one representative of 3 independent immunoblot assays. **B** HPLC-MS/MS quantification of the total ceramides, the specified ceramide species. Shown (mean ± SD) is one representative of three independent experiments that were quantified in triplicates. **C** With a final molar ratio of 6.5 PA into 1 FF-BSA, transfected cells were replaced with fresh medium containing 0, 50 or 250 μM of PA 48 h later for indicated periods. 0 μM represents the medium containing 0.25% of FF-BSA and NaOH. Shown is one representative of three independent experiments for determining the transcriptional levels of *CERS2*, *CERS6* and *DEGS1* in differently treated cells. Immunoblot analysis determined CERS2 and CERS6 when the cells were treated with 250 μM of PA for 24 h. **D** The cells (in triplicates) were treated with medium containing 250 μM PA alone, or inclusion of 5 μM Myr for 24 h and analysed using HPLC-MS/MS. The control medium contained 0.25% of FF-BSA, NaOH and DMSO. Shown is one representative of three independent experiments. Data (mean ± SD) represent the increased folds of total ceramides and the indicated ceramide species relative to that of empty-vector-transfected HepG2 cells cultured with control medium. The differences were compared with one-way ANOVA analysis. ^$^*P <* 0.05, ^$$^*P <* 0.01, ^$$$^*P <* 0.001 between Ref-HBV and empty-vector; *P < 0.05, **P < 0.01, ***P < 0.001 between Ref-HBV and mtPreS1 or mtPreS2; ^#^*P <* 0.05, ^##^*P <* 0.01, ^###^*P <* 0.001 between mtPreS1 and mtPreS2

Due to HBsAg retention within the hepatocyte ER, we examined the expression levels of genes related to ER stress. Protein kinase R-like ER kinase (PERK), inositol-requiring enzyme 1α (IRE1α) and activating transcription factor (ATF)-6 are anchored to the ER membrane and initiate three classical pathways to restore cellular protein homeostasis under ER stress conditions [33]. Compared with the results obtained after empty-vector transfection, transfection with mtPreS1, mtPreS2 or Ref-HBV significantly enhanced the gene expression levels of IRE1α in HepG2 cells. The expression of the ER chaperone protein GRP78 and transducer molecules in the IRE1α pathway, including XBP1 and CHOP, was upregulated in cells at both the transcriptional and translational levels after HBV transfection. Notably, compared with Ref-HBV-transfected HepG2 cells, mtPreS1- or mtPreS2-transfected cells expressed significantly higher levels of ER stress-related genes (Fig. 3A). However, no significant alteration in the transcriptional levels of PERK and ATF6 was detected after the HBV transfection (Additional file 1: Fig. S6). These results indicated that the IRE1α-XBP1 pathway, which is key to lipid metabolic homeostasis [33], responded mainly to HBsAg retention in the hepatocytes.

Abnormal accumulation of ceramides is a hallmark of metabolism-related disorders. Ceramides exert profoundly different effects depending on amide-linked fatty acid chain lengths which are regulated by CerS1–CerS6 and DES1*.* We then quantified the transcriptional levels of *CERS1–CERS6*, which encode six ceramide synthases, and *DEGS1*, which encodes DES1. After transfection with different HBV plasmids, HepG2 cells showed significantly elevated transcription of *CERS2*, *CERS6* and *DEGS1*, particularly in cells transfected with the PreS variants (Additional file 1: Fig. S7A). Using HPLC-MS/MS, we determined the amounts and species of ceramides with various lengths of fatty acyl chains. Compared with empty-vector-transfected cells, HepG2 cells generated significantly more ceramides after HBV transfection, particularly after transfection with mtPreS1 or mtPreS2. The level of the C16:0 ceramide species, the biosynthesis of which is mediated by CERS6 and CERS5, increased 3-fold after Ref-HBV transfection and 5-fold after transfection with either mtPreS1 or mtPreS2. The levels of C22-C24 ceramides, which are the major membrane components of hepatocytes and are regulated by CERS2, increased no more than 3-fold (Fig. 3B, Additional file 1: Table S5). CerS5 is predominantly expressed in the organs of the testis, epididymal white adipose tissue, lung, spleen and thymus [34]. Because the *CerS5* transcriptional level was not significantly increased after HBV transfection compared with that in empty-vector-transfected cells (Additional file 1: Fig. S7A), CERS6 and CERS2 might be the main mediators of increases in C16:0 and C22-C24 ceramide species.

### Free fatty acids aggravated the generation of abnormal ceramides in HBV-transfected cells

Modern diets are rich in unhealthy fats, and excess lipid exposure imposes significant challenges on the ER action [33]. We treated HBV-transfected HepG2 cells with variated PA concentrations to mimic the overload of saturated fatty acids. Compared with empty-vector-transfected HepG2 cells, the PA treatment led to significant elevations in the transcriptional levels of *CERS2*, *CERS6* and *DEGS1* in HBV-transfected cells. In particular, HepG2 cells transfected with either mtPreS1 or mtPreS2 exhibited higher expression of *CERS2*, *CERS6* and *DEGS1* than Ref-HBV-transfected cells. The effect depended on the concentration and time period of PA treatment (Fig. 3C). The transfection of Huh7 cell line and L02 cell line with three HBV plasmids resulted in the same outcomes (Additional file 1: Fig. S7B and S7C). The increase in CERS6 and CERS2 protein levels was confirmed by immunoblotting (Fig. 3C).

An HPLC-MS/MS analysis showed that the abundance of certain ceramide species in HepG2 cells without HBV transfection increased after PA treatment. Notably, significantly higher levels of ceramides were generated in HBV-transfected cells than in empty-vector-transfected cells that were treated with the same concentrations of PA, especially the cells that were transfected with PreS variants. Among that of the different ceramide species, the level of C16:0 ceramide was elevated the highest (Fig. 3D). The addition of Myr, a specific inhibitor of de novo ceramide synthesis, partially reversed the increase in abnormal ceramides (Fig. 3D and Additional file 1: Table S5). All these results indicated that the AA property alterations in HBV envelop proteins due to PreS mutations can inhibit HBsAg secretion from infected hepatocytes. HBsAg retention within the ER disturbed hepatocyte lipid metabolism homeostasis to generate abnormal amounts and abnormal species of ceramides.

### Infection with PreS variants and free fatty acids synergistically activated the liver macrophage NLRP3 through the abnormal ceramides

**Fig. 4:**
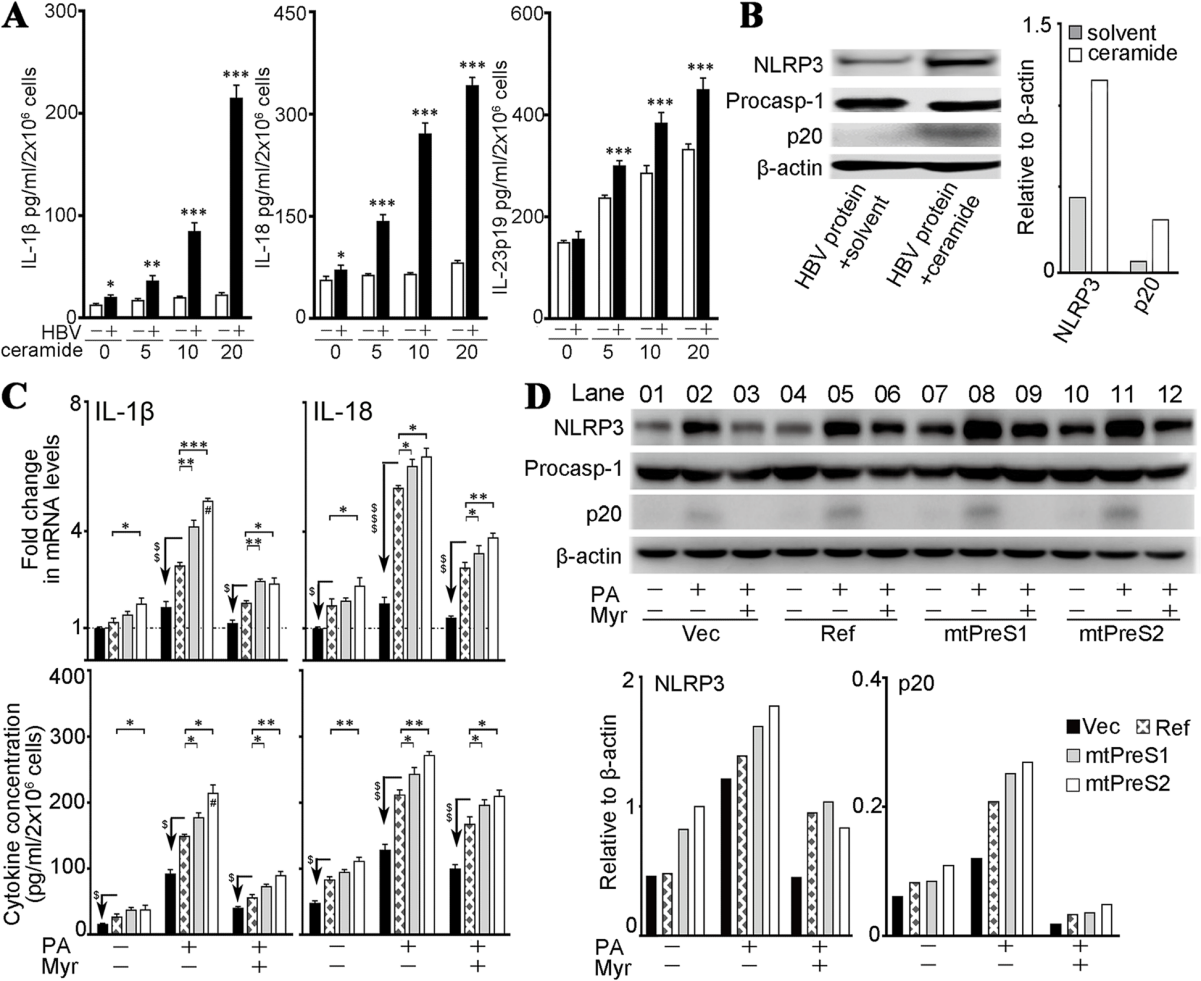
Activation of the macrophage NLRP3 inflammasome by ceramides from HBV-transfected hepatocytes. **A**, **B** Two million of murine peritoneal macrophages were stimulated with 5 μg/ml of HBV proteins alone, or addition of 0, 5, 10 or 20 μM of C6-ceramide for 24 h. 0 μM represents the medium containing 0.1% DMSO (solvent for C6-ceramide). **A** Bar graphs (mean ± SEM) show the amounts of secreted (in supernatant) IL-1β, IL-18 and IL-23 determined in three independent experiments. The differences were compared with paired two-tailed Student’s *t-*test. *P < 0.05, **P < 0.01 and ***P< 0.001 between the cells stimulated with specified concentration of C6-ceramide alone (empty columns) and those with addition of the HBV proteins. **B** The cells treated with the HBV proteins alone (HBV protein + solvent) or addition of 20 μM C6-ceramide (HBV protein + ceramide) were collected for analysis of NLRP3, procaspase-1 and cleaved caspase-1 (p20) generation. The immunoblot image shows one representative of three independent experiments, and the bar graph shows the relative amount of indicated proteins. **C, D** As described in Fig. 3D, the medium from differently treated HepG2 cells was collected and mixed with same volume of completed DMEM respectively as conditioned medium (CM) (depicted in Additional file 1: Fig. S8). Two million macrophages were treated with different CM for 72 h. **C** Bar graphs (mean ± SEM) show the macrophage generation of indicated cytokines determined by qRT-PCR (upper panel), or secretion in supernatant quantified by ELISA (low panel), from three independent experiments. The differences were compared with one-way ANOVA. ^$^*P <* 0.05, ^$$^*P <* 0.01, ^$$$^*P <* 0.001 between Ref-HBV and empty-vector; *P < 0.05, **P < 0.01, ***P < 0.001 between Ref-HBV and mtPreS1 or mtPreS2; ^#^*P <* 0.05 between mtPreS1 and mtPreS2. **D** Immunoblot analysis of NLRP3 expression and activation in the differently treated macrophages. Bar graphs show the relative amount of specified proteins of the experiment

Because of their interface in the liver, macrophages constantly receive the signals from hepatocytes. We tested the effects of hepatocyte-derived ceramides on the activation of the macrophage NLRP3 inflammasome. After stimulation with either HBV proteins or ceramides alone, the inflammatory macrophages secreted IL-1β, IL-18 and IL-23. However, the production of IL-1β and IL-18 was synergistically enhanced in the macrophages stimulated by HBV proteins in the presence of ceramides. This synergistic effect depended on the ceramide concentration (Fig. 4A). An immunoblot analysis showed that NLRP3 generation and activation, which is indicated by production of cleaved caspase-1 (p20), were augmented with the dual stimulation of HBV protein and ceramides (Fig. 4B).

To confirm the effects of abnormal ceramides derived from HBV-infected hepatocytes, we treated macrophages with conditioned medium (CM) from HepG2 cells that had been transfected with either an empty-vector or different HBV plasmids (depicted in Additional file 1: Fig. S8). Compared with those cultured in the CM of empty-vector-transfected HepG2 cells, the macrophages treated with the CM of HBV-transfected cells generated significantly higher levels of IL-1β and IL-18. Particularly, the CM of the PreS variant-transfected cells augmented the cytokine production in macrophages to a greater extent than the CM of the Ref-HBV-transfected cells (Fig. 4C). In addition, an immunoblot analysis showed that the CM of PreS variant-transfected cells enhanced the NLRP3 generation and p20 production in macrophages. When de novo ceramide synthesis in HBV-transfected cells was inhibited by myriocin, the stimulatory effect of CM on macrophage NLRP3 was attenuated (Fig. 4D).

### Infection with HBV PreS variants induced hepatic steatosis related to abnormal ceramide generation in the liver

**Fig. 5:**
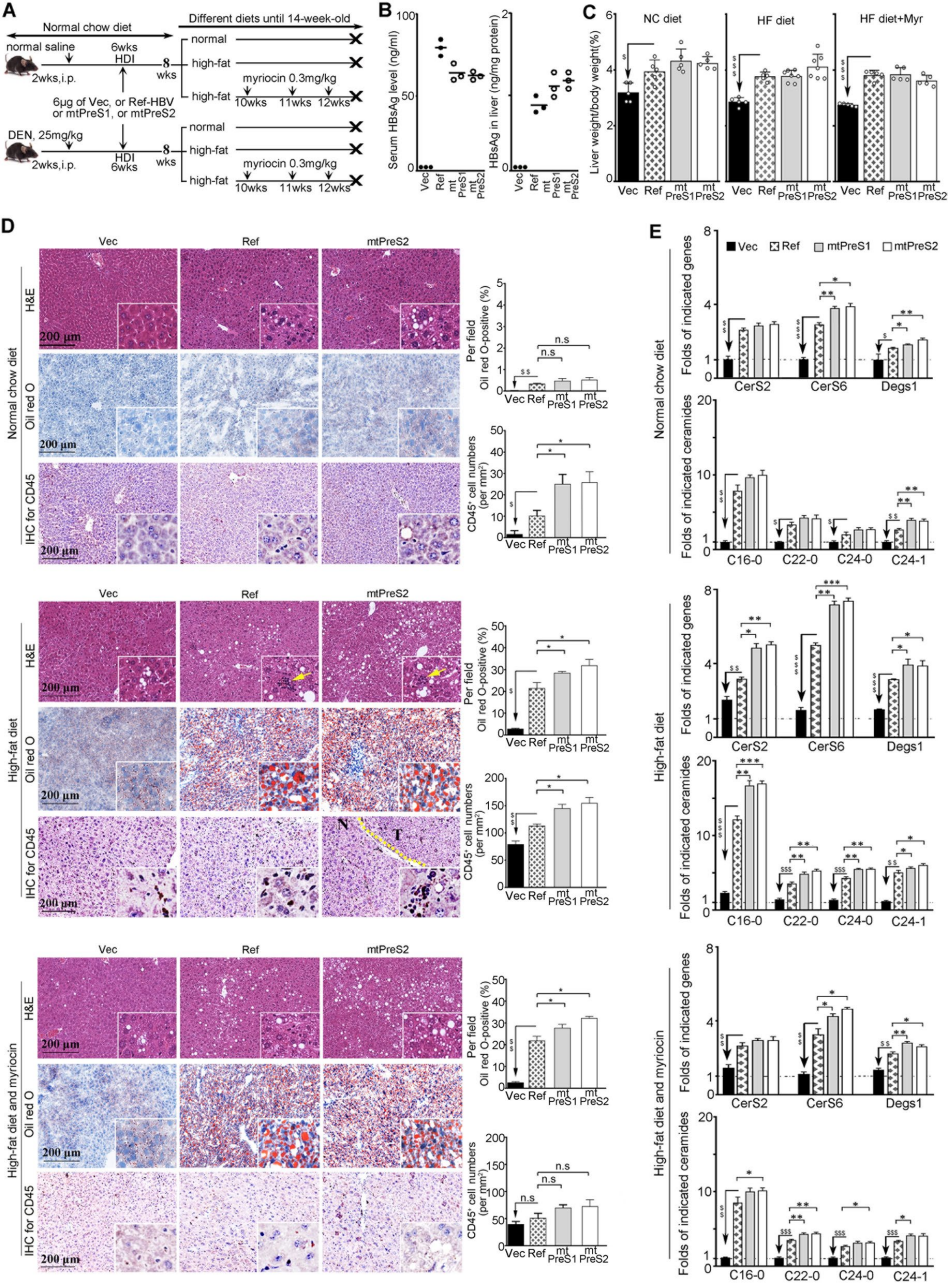
Hepatic steatosis and generation of different ceramide species in mice injected with different HBV plasmids. **A** Experiment scheme. All mice were sacrificed at their 14 weeks old. *n* = 5–7. **B** HBsAg detected in sera and in liver tissues of the mice 1 week after plasmid injection; each dot indicates one mouse. *n* = 3. **C** Ratios of liver weight to body weight of the mice fed different diets; each dot indicates one mouse. *n* = 5–7. **D** Representative images of H&E staining (upper), Oil red O staining (middle) and CD45 IHC staining (lower) of the indicated mouse liver sections. The others are in Additional file 1: Fig. S11. T, tumour; N, non-tumour. Bar graphs show the proportions of steatosis and the density of CD45-positive cells in each group. **E** From each group, 3 mice were removed and their tumour-free liver tissues were collected. Transcriptional levels of *CerS2*, *CerS6* and *Degs1* (upper panel) were determined by qRT-PCR. The amounts of C16:0 ceramide and C22-24 ceramides with different diets (low panel) were quantified by HPLC-MS/MS. Shown are changed fold (mean ± SEM) relative to the uninfected mice with NC diet. The differences were compared with one-way ANOVA. n.s. no statistical difference; ^$^*P <* 0.05, ^$$^*P <* 0.01, ^$$$^*P <* 0.001 between Ref-HBV and empty-vector; *P < 0.05, **P< 0.01, ***P < 0.001 between Ref-HBV and mtPreS1 or mtPreS2. No statistical difference was observed between mtPreS1 and mtPreS2

To investigate the impacts of the HBV PreS mutations that cause AA property alterations of envelop proteins in vivo, we infected the mouse livers through administering same amounts of empty-vector, Ref-HBV, mtPreS1 or mtPreS1 plasmids to male C57BL/6J mice by intravenous hydrodynamic injection. One week after plasmid injection, we removed 3 mice from each group to confirm the HBV transfection in the mouse livers. The mice receiving same HBV plasmid injection were allocated to two subgroups and fed NC or HF diets for 5 weeks (Fig. 5A). Compared with Ref-HBV-injected mice, mtPreS1- or mtPreS2-injected mice displayed relatively low serum HBsAg levels but higher HBsAg levels in the liver (Fig. 5B).

Beginning with different diet types, two subgroups of Ref-HBV-injected mice showed similar serum HBsAg levels. The two subgroups of mice that were injected with mtPreS1 or mtPreS2 also showed similar serum HBsAg levels (Additional file 1: Fig. S9). All mice were sacrificed at 14 weeks old, and the body weights of HF diet mice were greater than NC diet mice. With the same diet type, the body weights did not differ between the mice that were injected with empty-vector and those that were injected with Ref-HBV or mtPreS1 or mtPreS2 (Additional file 1: Fig. S10). However, compared with empty-vector-injected mice, all the mice injected with HBV plasmids presented with a greater ratio of liver weight to body weight, particularly those injected with PreS variants, including the NC diet mice (Fig. 5C).

In the H&E-stained liver sections prepared from mice injected with HBV plasmids and fed the NC diet, some lipid deposition was observed. We then performed Oil red O staining to detect the evidence of hepatic steatosis. Among the NC diet mice, the lipid deposition was detectable in the HBV plasmid-injected mouse livers, but did not significantly differ in the livers of mice that were injected with Ref-HBV and those that were injected with mtPreS1 or with mtPreS2. However, among the HF diet mice, the livers of mice that were injected with HBV plasmids showed aggravated steatosis compared to those with empty-vector, as indicated by larger areas stained with Oil red O. The numbers of infiltrated inflammatory cells, which were stained positively for leukocyte common antigen CD45, were higher in the HBV-injected mice than in the empty-vector-injected mice. Notably, the mice injected with either mtPreS1 or mtPreS2 presented more severe steatosis and inflammation than those injected with Ref-HBV (Fig. 5D and Additional file 1: Fig. S11). We then analysed the expression of the *CerS2*, *CerS6* and *Degs1* genes, which regulate the generation of different ceramide species, in the mouse livers. Compared with those in the empty-vector-injected mouse livers, the transcriptional levels of these genes were significantly elevated in the HBV plasmid-injected mice, particularly in the mice injected with PreS mutants, regardless of diet type (Fig. 5E). An HPLC-MS/MS analysis showed that the total ceramide amounts increased and that the C16:0 ceramide level was the most highly elevated among different species of ceramides, although the levels of C22-24 ceramides also increased. When the mice were fed the HF diet, *CerS2*, *CerS6* and *Degs1* transcription and ceramide generation were further enhanced. The elevation of C16:0 ceramide abundance was the most significant (Fig. 5E and Additional file 1: Fig. S12).

We intraperitoneally injected myriocin to inhibit de novo ceramide synthesis in the HF-fed mice. The liver weight/body weight rate showed no significant reduction (Fig. 5C). However, the gene expression of *CerS2*, *CerS6* and *Degs1* was decreased, and the increase in C16:0 ceramide and C22-24 ceramide levels was partially decreased (Fig. 5E). The infiltration of inflammatory cells was attenuated (Fig. 5D).

### HBV PreS variant injection and a HF diet synergistically promoted autochthonous HCC development associated with abnormal ceramide generation

**Fig. 6:**
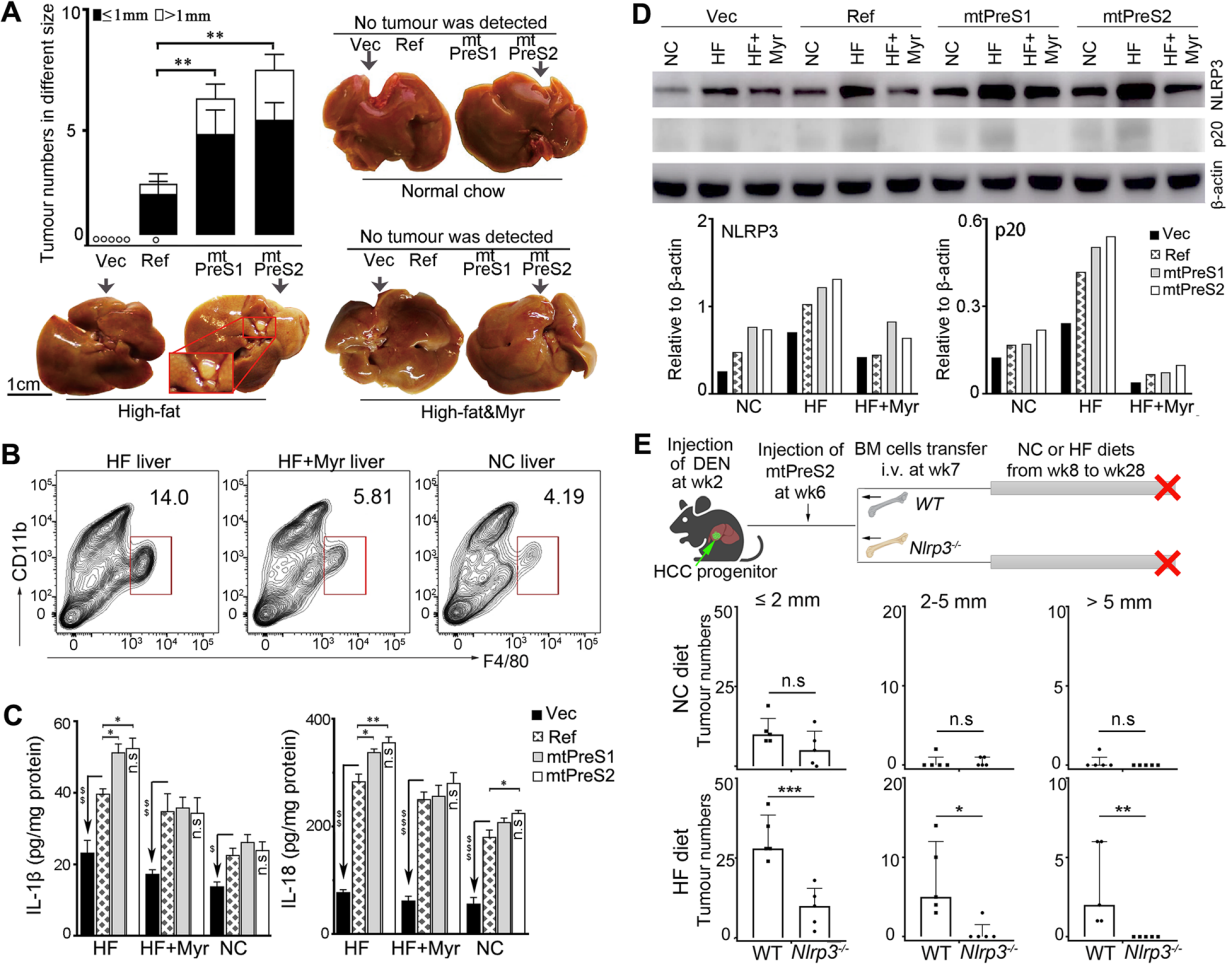
NLRP3 activation in liver macrophages on development of murine autochthonous HCC. **A** The bar graph (median with IQR) shows tumour numbers in variated sizes of different plasmid-injected mice fed the HF diet, *n* = 5–7. Images show the representative mouse livers with different diets after injection of empty-vector and mtPreS2. The others are in Additional file 1: Fig. S13. **B** FCM profiles of liver inflammatory macrophages isolated from tumour-free liver tissues in the group of mice that were injected with mtPreS2-plasmid and fed with indicated diets. **C** Bar graphs (mean ± SEM) show the IL-1β and IL-18 levels in the liver intercellular fluid of each mouse. **D** Intrahepatic cells were purified through 25%/50% Percoll gradient centrifugation, pooled together from one group of mice with same treatment for determination of NLRP3 and the cleaved caspase-1 (p20) by immunoblotting. Bar graphs show the relative amount of specified proteins. **E** DEN-injected mice received 6 μg of mtPreS2 plasmids. One week later, the mice (receipts) were irradiated with 4 Gy of X-ray for depletion of host immune cells. One subgroup of receipts obtained bone marrow (BM) cells from the *Nlrp3*^*−/−*^ mice, and another obtained BM cells from C57BL/6J (WT). Different diets began at their 8 weeks old until 28 weeks old. Bar graphs (median with IQR) show the tumour numbers in variated sizes fed the NC diet (upper panel) or HF diet (low panel), *n* = 5, each dot represents one mouse. Tumour numbers in differently treated mice (**A**, **E**) were compared with the Mann-Whitney *U* test. The concentration of cytokines in the mouse livers with different treatments was compared with the one-way ANOVA analysis. ^$^*P <* 0.05, ^$$^*P <* 0.01, ^$$$^*P <* 0.001 between Ref-HBV and empty-vector; *P < 0.05, **P < 0.01, ***P < 0.001 between Ref-HBV and mtPreS1 or mtPreS2 or between WT and *Nlrp3*^*−/−*^. n.s. no statistical difference

Before different HBV plasmids were injected, some mice were given DEN by intraperitoneal injection to induce HCC progenitor cells [15] (Fig. 5A). We detected liver tumour nodules only in the mice that were injected with HBV plasmids and fed the HF diet. The tumour loads were higher in the mice injected with PreS mutants than in those injected with Ref-HBV (Fig. 6A). When the mice were injected with HBV plasmids and fed the NC diet, no mouse developed tumour. Moreover, no tumour was observed in the empty-vector-injected mice fed the HF diet (Additional file 1: Figs. S11B and S13). Additionally, as determined upon sacrifice at 14 weeks old, none of the mice developed liver tumour regardless of their diet types after HBV plasmid injection without DEN injection (Additional file 1: Fig. S14). To validate the effect of the abnormal ceramides on HCC development, a subset of mice fed the HF diet received myriocin treatment (Fig. 5A). No tumours were detected in mice after ceramide de novo synthesis was inhibited (Fig. 6A). These results indicated that hepatocyte infection with HBV, particularly with PreS variants, synergistically promoted HCC development in the context of HF diet. The synergistic effect of HF diet and HBV infection on HCC promotion was associated with abnormal ceramide generation.

### HF diet promoted inflammatory macrophage infiltration and activation in livers of the mice infected with HBV PreS variants

Because of the evidence of inflammatory cell infiltration (Fig. 5D), we analysed the effect of different diet types on the infiltration of inflammatory macrophages in the mouse livers. Intrahepatic infiltrating cells were prepared from tumour-free liver tissues of mtPreS2-injected mice that were fed different diets. A FCM analysis showed that the HF diet tripled the infiltration of CD11b^+^F4/80^+^ inflammatory macrophages compared with the number of infiltrating macrophages in mice fed the NC diet (Fig. 6B). Moreover, we detected significantly higher amounts of IL-1β and IL-18 in the livers of mice fed the HF diet than in those fed the NC diet (Fig. 6C). Myriocin administration in mice fed the HF diet alleviated macrophage infiltration and inflammatory cytokine generation in the liver (Fig. 6B, C).

We then purified the liver macrophages through 25%/50% Percoll gradient centrifugation. An immunoblot analysis showed that liver macrophages in the PreS variant-injected mice activated macrophage NLRP3, including in the NC diet fed mice. Notably, the macrophages in the HBV-injected mice generated more NLRP3 and caused higher levels of NLRP3 activation that was indicated by more amounts of p20 production after HF diet, especially the macrophages in the mice injected with the PreS variants. Myriocin administration attenuated NLRP3 activation in mice fed the HF diet (Fig. 6D).

### Blocking NLRP3 activation of inflammatory macrophages significantly reduced autochthonous HCC development

Hepatocytes also express NLRP3. To confirm the effects of NLRP3 in liver inflammatory macrophages on autochthonous HCC development, we transferred bone marrow cells obtained from *Nlrp3*^*−/−*^ mice into mtPreS2-injected mice. The mice were fed different diet types for 20 weeks after bone marrow cell transfer. The liver tumour loads were significantly reduced in the mice that received the *Nlrp3*^*¬/−*^ bone marrow compared with those in the mice that received C57BL/6J bone marrow cells. The reduction of liver tumour loads in the *Nlrp3*^*−/−*^ bone marrow receipt mice was more profound in those that were fed the HF diet than in those that were fed the NC diet (Fig. 6E). These results indicated the importance of NLRP3 inflammasome in liver macrophages for promoting autochthonous HCC development.

## Discussion

In this study, we analysed 35 of 236 HBsAg-seronegative HCC patients and found that nonneoplastic liver tissues of these patients harboured HBV cccDNA, which carried extensive mutations in PreS/S regions compared with a reference HBV isolate that was obtained from one HBsAg-seropositive HCC patient. Most of the variants presented with altered AA properties of envelope proteins. We constructed the Ref-HBV plasmid that contained 1.3×genome of the HBV isolate from one HBsAg-seropositive HCC patient and plasmid of mtPreS1 that was mutated only in the PreS1 and mtPreS2 that was mutated only in the PreS2, considering the PreS mutations in 35 HBsAg-seronegative HCC patients. Both the plasmids of mtPreS1 and mtPreS2 shared identical sequences of S and other regions with Ref-HBV plasmid. After transfection with three different plasmids, we detected similar levels of synthesized HBsAg by the cells. However, HBsAg secretion from the hepatocytes that were transfected with mtPreS1 or mtPreS2 was significantly inhibited compared with that transfected with Ref-HBV. HBsAg was mainly restrained in the ER compartment that disturbed the lipid metabolic homeostasis. HBV-transfected hepatocytes generated abnormal ceramides both in terms of amounts and species, and this effect was aggravated by free fatty acid overload, particularly the hepatocytes transfected with PreS mutants. NLRP3 inflammasome in the liver inflammatory macrophages was synergistically activated by HBV proteins and hepatocyte-derived ceramides, depending on the ceramide concentration. After injection of same amounts of empty-vector, Ref-HBV, mtPreS1 or mtPreS2 into DEN-treated male C57BL/6J mice to cause liver HBV infection, we detected liver tumour nodules only in the mice that were fed the HF diet. Particularly, the tumour loads were higher in the mice injected with mtPreS1 or mtPrS2 than in those injected with Ref-HBV. Inhibition of de novo ceramide synthesis or blocking NLRP3 activation in liver macrophages led to HCC development repression. Therefore, the alteration of AA properties in HBV envelope proteins caused HBsAg retention within the infected hepatocyte ER could promote the HCC development (Additional file 1: Fig. S15). The significance of chronic inflammation in cancer development has been previously highlighted [35]. Therefore, reducing the generation of abnormal ceramides in the liver or inhibiting NLRP3 activation in liver macrophages may be helpful for HBV-infected individuals to repress HCC development.

It has been documented that in-frame deletions or introduction of a stop codon in PreS/S regions resulted in HBsAg retention within hepatocyte ER to promote HCC via multiple pathways involved in ER stress [10]. Our current study indicated that the alteration of AA properties in HBV envelope proteins might change the viral transmembrane topology to cause HBsAg retention within the infected hepatocytes. Although some synthesized HBsAg was observed in the mitochondria of the HBV plasmid-transfected cells, we detected HBsAg mainly localized to the hepatocyte ER. HBV virion assembly/secretion occurs in the hepatocyte ER [8, 9], where ceramides are generated [18]. Hepatocytes with HBsAg retention displayed dysfunctional lipid metabolism to generate a higher level of abnormal ceramide amounts and ceramide species. The synthesis of C16:0 ceramide was the most notably increased, and this increase was enhanced in cells with excessive saturated fatty acids, which correlated with the increase in ceramide synthase CerS6 expression. Elevated levels of C22-C24 ceramides were also observed, the generation of which is regulated by CerS2. In experimental *CerS2-null* mice, which have been reported to be prone to HCC, the elevation of C16:0 ceramide species was also reported [36]. However, the elevation of C22-C24 ceramide levels was not found in *CerS2-null* mice. We detected C16:0 ceramide was the most notably increased in cultured hepatocytes and in mouse livers after HBV transfection, especially PreS variant transfection. The tumour loads were higher in the mice injected with PreS variants than in mice injected with Ref-HBV which was derived from one HBsAg-seropositive HCC patient and had normal HBsAg secretion. These results indicated that C16:0 ceramide might be an important mediator in promoting HCC in individuals with OBI in a high-fat diet context.

The integration of HBV breakpoints, mainly at viral X and Precore/Core genes, can drive the hepatocyte oncogenic transformation [9, 16, 17]. However, HBV cccDNA were mainly detected in the nonneoplastic liver tissues of the HBsAg-seronegative HCC patient samples that carried extensive mutations in the PreS/S region. Previous studies have shown that OBI can lead to severe liver diseases, including HCC, in certain situations [8, 14]. Our experiments with autochthonous HCC murine models indicated that only HBV-injected mice fed the HF diet, not the NC diet, developed tumours. Without HBV injection, however, no mice developed liver tumours regardless of diet types. In a cohort consisting of 13,032 employees of a company in China, a 15-year follow-up study reported that the patients with non-alcoholic fatty liver disease developed mainly diabetes, hypertension or hyperuricaemia, and none progressed into severe liver disease without HBV infection [37]. Our current results indicated a synergistic effect of a HF diet and OBI on HCC development. The HF diet is an important cofactor that enhances the generation of abnormal ceramide to induce chronic liver inflammation for HCC promotion [1, 9, 15, 24, 35].

Many OBIs are associated with PreS variant infections, including in some adults who received the HBV vaccine at infancy [8, 12, 38]. MAFLD is becoming the most common cause of chronic liver diseases and an important risk factor for HCC. The cause of MAFLD is heterogeneous and influenced by multiple factors, including dietary pattern [1, 4]. To reduce HCC risk, therefore, individuals with PreS variant-related OBI should prevent metabolic disorders by, at least, improving their diet pattern and receive proper therapy after metabolic disorders manifest. NLRP3 activation can be triggered by various endogenous “danger signals”, and the ceramide is one of the most important “danger signals” to mediate the development of metabolism-related disorders and liver disease progression [18, 19]. Due to the interplay between hepatocytes and macrophages in the liver, we found that HBV proteins together with ceramides coactivated the NLRP3 inflammasome to enhance liver inflammation. By transferring the bone marrow cells from the mice of *Nlrp3*^*−/−*^ or *wild-type* into mtPreS2-injectived mice, we validated the crucial effects of macrophage NLRP3 in promoting HCC. It has been documented that a small-molecule NLRP3 inhibitor, MCC950, reduced liver inflammation and fibrosis in experimental mice with fatty liver diseases [19]. We found that in both our cell culture and mouse model systems, HBsAg retention within the hepatocyte ER disturbed lipid metabolism, leading to abnormal ceramide generation, which in turn coactivated liver macrophages with HBV proteins. Reducing ceramide generation or blocking NLRP3 activation with this NLRP3 inhibitor during antiviral therapy may be helpful in repressing HCC development in HBV-infected patients carrying PreS mutations. However, the effectiveness of NLRP3 inhibitors in reducing HCC risk needs to be confirmed.

## Conclusions

Mutations in HBV PreS1 or PreS2 altered the AA properties of envelope proteins and inhibited HBsAg secretion from infected hepatocytes. PreS variant-infected hepatocytes generated abnormal amounts and abnormal species of ceramides that coactivated the NLRP3 inflammasome in liver macrophages to promote autochthonous HCC development. Inhibition of abnormal ceramide generation or blockade of NLRP3 activation may repress HCC development after OBI infection.

## Supplementary Information

**Additional file 1: Supplementary Methods**. **Tables S1**. Primers used for detection of HBV cccDNA, amplification and sequencing of PreS/S regions. **Tables S2**. Antibodies and ELISA kits. **Tables S3**. Primers used in qRT-PCR analysis. **Tables S4**. Histology-confirmed 1823 HCC patients with different serologically viral markers. **Tables S5**. The amounts of specified ceramide species determined by HPLC-MS/MS in HepG2 cells that were differently treated for 24 h after HBV plasmid transfection. **Fig. S1**. Analysis of HBV PreS/S regions in 35 HCC patients with serological markers of HBsAg(-) & anti-HBc(+) & anti-HCV(-). **Fig. S2**. Aliment of 35 HBV isolates from the HBsAg-seronegative HCC patients. **Fig. S3**. Profile of the HBV replicating plasmids. **Fig. S4**. The staining of envelop proteins and hepatocyte ER in five independent experiments. **Fig. S5**. HBsAg secretion from hepatocytes after transfection with different HBV plasmids. **Fig. S6**. Transcriptional levels of *IRE1α, ATF6*, and P*ERK* in HepG2 cells after transfection with different HBV plasmids. **Fig. S7**. Transcriptional levels of *CERS1-6* and *DEGS1.* **Fig. S8**. Schematic diagram of culture system to examine the effects of ceramides from hepatocytes that were transfected with different HBV plasmids on inflammatory macrophages. **Fig. S9**. Serum levels of HBsAg in mice that were injected with different HBV plasmids. **Fig. S10**. Body weight and liver weight of the mice fed different diet types. **Fig. S11**. Representative histological images of the DEN-treated mice livers with different diet types. **Fig. S12**. Transcriptional levels of *CerS1-6* and *Degs1* genes in mice livers. **Fig. S13**. Liver macroscopic appearance and tumour numbers in the DEN-treated mice with different diet types. **Fig. S14**. Liver weight and H&E staining of mice livers without DEN injection. **Fig. S15**. Graphic abstract.

## Data Availability

All the data supporting the findings of this study are available within the article and its additional files.

## Abbreviations

AA: Amino acid
ATF-6: Activating transcription factor-6
BSA: Bovine serum albumin
cccDNA: Covalently closed circular DNA
CERS: Ceramide synthase
DEN: Diethylnitrosamine
DES1: Dihydroceramide desaturase-1
ER: Endoplasmic reticulum
FCM: Flow cytometry
HBsAg: Hepatitis B surface antigen
HBV: Hepatitis B virus
HCC: Hepatocellular carcinoma
HDI: Hydrodynamic injection
HPLC-MS/MS: High-performance liquid chromatography coupled to tandem mass spectrometry
IHC: Immunochemistry
IRE1α: Inositol-requiring enzyme 1α
NLRP3: NOD-like receptor protein 3
OBI: Occult HBV infection
PA: Palmitic acids
PERK: Protein kinase R-like ER kinase
qRT-PCR: Quantitative real-time PCR
rcDNA: Relaxed circular DNA

## References

[1] Kulik L, El-Serag HB (2019). Epidemiology and management of hepatocellular carcinoma. Gastroenterology.

[2] Maucort-Boulch D, de Martel C, Franceschi S, Plummer M (2018). Fraction and incidence of liver cancer attributable to hepatitis B and C viruses worldwide. Int J Cancer.

[3] Makarova-Rusher OV, Altekruse SF, McNeel TS, Ulahannan S, Duffy AG, Graubard BI, Greten TF, McGlynn KA (2016). Population attributable fractions of risk factors for hepatocellular carcinoma in the United States. Cancer.

[4] Eslam M, Sanyal AJ, George J (2020). International Consensus P: MAFLD: a consensus-driven proposed nomenclature for metabolic associated fatty liver disease. Gastroenterology.

[5] Zeng J, Qin L, Jin Q, Yang RX, Ning G, Su Q (2022). Prevalence and characteristics of MAFLD in Chinese adults aged 40 years or older: a community-based study. Hepatobiliary Pancreat Dis Int..

[6] Yu MW, Lin CL, Liu CJ, Yang SH, Tseng YL, Wu CF (2017). Influence of metabolic risk factors on risk of hepatocellular carcinoma and liver-related death in men with chronic hepatitis B: a large cohort study. Gastroenterology.

[7] Choe JW, Hyun JJ, Kim B, Han KD. Influence of metabolic syndrome on cancer risk in HBV carriers: a nationwide population based study using the National Health Insurance Service Database. J Clin Med. 2021;10(11):2401. 10.3390/jcm10112401 PMC8198770 34072289

[8] Raimondo G, Locarnini S, Pollicino T, Levrero M, Zoulim F, Lok AS (2019). Taormina Workshop on Occult HBVIFM: Update of the statements on biology and clinical impact of occult hepatitis B virus infection. J Hepatol.

[9] Seeger C, Mason WS (2015). Molecular biology of hepatitis B virus infection. Virology.

[10] Pollicino T, Cacciola I, Saffioti F, Raimondo G (2014). Hepatitis B virus PreS/S gene variants: pathobiology and clinical implications. J Hepatol.

[11] Qu C, Chen T, Fan C, Zhan Q, Wang Y, Lu J, Lu LL, Ni Z, Huang F, Yao H (2014). Efficacy of neonatal HBV vaccination on liver cancer and other liver diseases over 30-year follow-up of the Qidong Hepatitis B Intervention Study: a cluster randomized controlled trial. PLoS Med.

[12] Wang R, Liu C, Chen T, Wang Y, Fan C, Lu L, Lu F, Qu C (2021). Neonatal hepatitis B vaccination protects mature adults from occult virus infection. Hepatol Int.

[13] Xu L, Wei Y, Chen T, Lu J, Zhu CL, Ni Z, Huang F, Du J, Sun Z, Qu C (2010). Occult HBV infection in anti-HBs-positive young adults after neonatal HB vaccination. Vaccine.

[14] Azuma M, Ebihara T, Oshiumi H, Matsumoto M, Seya T (2012). Cross-priming for antitumor CTL induced by soluble Ag + polyI:C depends on the TICAM-1 pathway in mouse CD11c(+)/CD8alpha(+) dendritic cells. Oncoimmunology.

[15] He G, Dhar D, Nakagawa H, Font-Burgada J, Ogata H, Jiang Y, Shalapour S, Seki E, Yost SE, Jepsen K (2013). Identification of liver cancer progenitors whose malignant progression depends on autocrine IL-6 signaling. Cell.

[16] Villanueva A (2019). Hepatocellular carcinoma. N Engl J Med.

[17] Zhao LH, Liu X, Yan HX, Li WY, Zeng X, Yang Y, Zhao J, Liu SP, Zhuang XH, Lin C (2016). Genomic and oncogenic preference of HBV integration in hepatocellular carcinoma. Nat Commun.

[18] Turpin-Nolan SM, Bruning JC (2020). The role of ceramides in metabolic disorders: when size and localization matters. Nat Rev Endocrinol.

[19] Mridha AR, Wree A, Robertson AAB, Yeh MM, Johnson CD, Van Rooyen DM, Haczeyni F, Teoh NC, Savard C, Ioannou GN (2017). NLRP3 inflammasome blockade reduces liver inflammation and fibrosis in experimental NASH in mice. J Hepatol.

[20] Tian D, Qiu Y, Zhan Y, Li X, Zhi X, Wang X, Yin L, Ning Y (2012). Overexpression of steroidogenic acute regulatory protein in rat aortic endothelial cells attenuates palmitic acid-induced inflammation and reduction in nitric oxide bioavailability. Cardiovasc Diabetol.

[21] Perdoni F, Signorelli P, Cirasola D, Caretti A, Galimberti V, Biggiogera M, Gasco P, Musicanti C, Morace G, Borghi E (2015). Antifungal activity of Myriocin on clinically relevant Aspergillus fumigatus strains producing biofilm. BMC Microbiol.

[22] Morad SAF, Ryan TE, Neufer PD, Zeczycki TN, Davis TS, MacDougall MR, Fox TE, Tan S-F, Feith DJ, Loughran TP (2016). Ceramide-tamoxifen regimen targets bioenergetic elements in acute myelogenous leukemia. J Lipid Res.

[23] Zhang X, Goncalves R, Mosser DM (2008). The isolation and characterization of murine macrophages. Curr Protoc Immunol.

[24] Zang M, Li Y, He H, Ding H, Chen K, Du J, Chen T, Wu Z, Liu H, Wang D (2018). IL-23 production of liver inflammatory macrophages to damaged hepatocytes promotes hepatocellular carcinoma development after chronic hepatitis B virus infection. Biochim Biophys Acta Mol Basis Dis.

[25] Li X, Xie T, Gao L, Ma C, Yang X, Liang X (2018). Prostaglandin E2 facilitates hepatitis B virus replication by impairing CTL function. Mol Immunol.

[26] Chen K, Wu Z, Zhao H, Wang Y, Ge Y, Wang D, Li Z, An C, Liu Y, Wang F (2020). XCL1/glypican-3 fusion gene immunization generates potent antitumor cellular immunity and enhances anti-PD-1 efficacy. Cancer Immunol Res.

[27] Caviglia GP, Abate ML, Tandoi F, Ciancio A, Amoroso A, Salizzoni M, Saracco GM, Rizzetto M, Romagnoli R, Smedile A (2018). Quantitation of HBV cccDNA in anti-HBc-positive liver donors by droplet digital PCR: a new tool to detect occult infection. J Hepatol.

[28] Da Costa LS, Arnoult D (2017). Organelle separation and cell signaling. Methods Mol Biol.

[29] Qu F, Zheng SJ, Liu S, Wu CS, Duan ZP, Zhang JL (2014). Serum sphingolipids reflect the severity of chronic HBV infection and predict the mortality of HBV-acute-on-chronic liver failure. PLoS One.

[30] Yu X, Lan P, Hou X, Han Q, Lu N, Li T, Jiao C, Zhang J, Zhang C, Tian Z (2017). HBV inhibits LPS-induced NLRP3 inflammasome activation and IL-1beta production via suppressing the NF-kappaB pathway and ROS production. J Hepatol.

[31] Wang M, Wang Y, Feng X, Wang R, Wang Y, Zeng H, Qi J, Zhao H, Li N, Cai J (2017). Contribution of hepatitis B virus and hepatitis C virus to liver cancer in China north areas: experience of the Chinese National Cancer Center. Int J Infect Dis.

[32] Liu S, Zhang H, Gu C, Yin J, He Y, Xie J, Cao G (2009). Associations between hepatitis B virus mutations and the risk of hepatocellular carcinoma: a meta-analysis. J Natl Cancer Inst.

[33] Lemmer IL, Willemsen N, Hilal N, Bartelt A (2021). A guide to understanding endoplasmic reticulum stress in metabolic disorders. Mol Metab.

[34] Gosejacob D, Jager PS, Vom Dorp K, Frejno M, Carstensen AC, Kohnke M, Degen J, Dormann P, Hoch M (2016). Ceramide synthase 5 is essential to maintain C16:0-ceramide pools and contributes to the development of diet-induced obesity. J Biol Chem.

[35] Hanahan D, Weinberg RA (2011). Hallmarks of cancer: the next generation. Cell.

[36] Pewzner-Jung Y, Brenner O, Braun S, Laviad EL, Ben-Dor S, Feldmesser E, Horn-Saban S, Amann-Zalcenstein D, Raanan C, Berkutzki T (2010). A critical role for ceramide synthase 2 in liver homeostasis: II. Insights into molecular changes leading to hepatopathy. J Biol Chem.

[37] Tang X, Weng H, Huang T (2021). Does NAFLD really lead to end-stage liver disease?. Translational Medical Research: Molecular mechanisms driving hepatocarcinogenesis in cirrhotic liver.

[38] Wu TW, Lin HH, Wang LY (2013). Chronic hepatitis B infection in adolescents who received primary infantile vaccination. Hepatology.

