# Supplementary material for: Occult infection with hepatitis B virus PreS variants synergistically promotes hepatocellular carcinoma development in a high-fat diet context by generating abnormal ceramides
Source: BMC Med. 2022 Sep 5;20:279. doi: 10.1186/s12916-022-02481-3 (PMC9442965; doi:10.1186/s12916-022-02481-3)
Supplement: Supplementary file 1 — Additional file 1: Supplementary Methods. Tables S1. Primers used for detection of HBV cccDNA, amplification and sequencing of PreS/S regions. Tables S2. Antibodies and ELISA kits. Tables S3. Primers used in qRT-PCR analysis. Tables S4. Histology-confirmed 1823 HCC patients with different serologically viral markers. Tables S5. The amounts of specified ceramide species determined by HPLC-MS/MS in HepG2 cells that were differently treated for 24 h after HBV plasmid transfection. Fig. S1. Analysis of HBV PreS/S regions in 35 HCC patients with serological markers of HBsAg(-) & anti-HBc(+) & anti-HCV(-). Fig. S2. Aliment of 35 HBV isolates from the HBsAg-seronegative HCC patients. Fig. S3. Profile of the HBV replicating plasmids. Fig. S4. The staining of envelop proteins and hepatocyte ER in five independent experiments. Fig. S5. HBsAg secretion from hepatocytes after transfection with different HBV plasmids. Fig. S6. Transcriptional levels of IRE1α, ATF6, and PERK in HepG2 cells after transfection with different HBV plasmids. Fig. S7. Transcriptional levels of CERS1-6 and DEGS1. Fig. S8. Schematic diagram of culture system to examine the effects of ceramides from hepatocytes that were transfected with different HBV plasmids on inflammatory macrophages. Fig. S9. Serum levels of HBsAg in mice that were injected with different HBV plasmids. Fig. S10. Body weight and liver weight of the mice fed different diet types. Fig. S11. Representative histological images of the DEN-treated mice livers with different diet types. Fig. S12. Transcriptional levels of CerS1-6 and Degs1 genes in mice livers. Fig. S13. Liver macroscopic appearance and tumour numbers in the DEN-treated mice with different diet types. Fig. S14. Liver weight and H&E staining of mice livers without DEN injection. Fig. S15. Graphic abstract. [file 12916_2022_2481_MOESM1_ESM.docx]

**Additional file for**

**Occult infection with hepatitis B virus PreS variants synergistically promotes hepatocellular carcinoma development in a high-fat diet context by generating abnormal ceramides**

Chang Liu^#^, Kun Chen^#^, Fei Zhao, Lingling Xuan, Yuting Wang, Chungui Xu, Zhiyuan Wu, Dongmei Wang, and Chunfeng Qu^*^

Supplementary **Methods**

**Preparation of solution**

1) Palmitic acid solution was prepared as previously described by Tian [20]. Briefly, palmitic acid (PA) (Sigma-Aldrich, St Louis, MO, USA) was dissolved into 0.1 M NaOH at 70°C to prepare the 1 M stock solution. The working solution was10 mM that was diluted with filter-sterilized 10% (w/v) of fatty-acid free bovine serum albumin (FF-BSA). The final molar ratio of PA to FF-BSA was 6.5:1 in cell culture. The control solution contained NaOH and 0.25% of FF-BSA.

2) Myriocin (Sigma-Aldrich) was dissolved in dimethyl sulfoxide (DMSO) to a final concentration of 20 mM stock. For cell culture, the stock solution was diluted into DMEM medium to a final concentration of 5 μM. Control solution contained 0.025% of DMSO and labeled as solvent. For intraperitoneal injection in animal experiments, the solution was prepared with normal saline from stock before usage every week. The final concentration was 75 μM, equaling 0.03 mg/ml. The dose administrated to each mouse was 0.3 mg/kg body weight. Mice in control group were intraperitoneally injected with the same volume of normal saline containing 0.375% of DMSO [21].

3) C6-ceramide (Sigma-Aldrich) was dissolved in DMSO to a final concentration of 20 mM stock [22]. The control solution contained 0.01% of DMSO and labeled as solvent.

4) Thioglycolate powder (Sigma-Aldrich) was dissolved in distilled water to a final concentration of 4% (w/v). After being autoclaved, the solution was stored in the dark at room temperature [23].

5) Diethylnitrosamine (DEN) (Sigma-Aldrich) was dissolved in sterilized normal saline to a final concentration of 50 mg/ml stock and stored at 4℃ in the dark. Before intraperitoneal injection in animal experiments, the stock solution was diluted to a final concentration of 1.5 mg/ml in normal saline. The dose administrated to each mouse was 25 mg/kg [15,24].

The information of antibodies used in IHC, Immunofluorescent, Immunoblotting and flow cytometry is listed in **Table S2**.

**Immunohistochemistry (IHC)**

Standard laboratory protocols were used. Human HCC samples and mice liver samples were sectioned, deparaffinized, and treated in 0.01 M citrate buffer (pH 6.0) at 96 °C for 15 min, followed by 3% hydrogen peroxide for 15 min. The sections were blocked with 10% of normal goat serum for 60 min at room temperature and then incubated with 1:400 diluted mouse anti-HBsAg (H2F4) monoclonal antibody (Bioss, Beijing, China) or incubated with 1:1000 diluted rabbit anti-mouse CD45 polyclonal antibody (Bioss) at 4°C overnight. After staining with horseradish peroxidase (HRP)-labeled anti-mouse IgG or HRP-labeled goat anti-rabbit IgG at room temperature for 30 min, the sections were colored with 3, 3’-diaminobenzidine (DAB) solution for 2-5 min (all reagents from ZSGB-Bio, Beijing, China). Hematoxylin staining was finally processed for nuclei staining. The sections were scanned and analysed using Aperio ScanScope software (Aperio Technologies).

**Immunofluorescent microscopy**

In the 8-well chamber slides, 8×10^4^/well of HepG2 cells were transfected with 300 ng HBV plasmids, the profile of which is in Fig. S3, and cultured for 48 h. To detect the colocalization of HBsAg with endoplasmic reticulum (ER), the cells were fixed with 4% of paraformaldehyde solution for 15 min. The cells were double stained with mouse anti-HBsAg (Bioss) and goat anti-human GRP78 (Santa Cruz), or rabbit anti-human calnexin (Proteintech) primary antibodies at 4 °C overnight. Then FITC-labeled anti-mouse IgG and Cy3-labeled anti-goat IgG, or Cy3-labeled anti-rabbit IgG were added and incubated for 1 h at room temperature. The slides were mounted with Vectashield for nuclei staining. To detect the colocalization of HBsAg with mitochondria, 48 h after plasmid transfection, HBV-plasmid-transfected cells were stained with prewarmed 200 nM of Mito-Tracker Red CMXRos (Beyotime) solution at 37 °C for 15 min. The cells were then fixed with 4% of paraformaldehyde solution for 15 min. After incubation in PBS containing 0.2% Triton X-100 for 10 min, the cells were then stained with mouse anti-HBsAg (Bioss) primary antibody at 4 °C overnight, followed with FITC-labeled anti-mouse IgG at room temperature for 1 h. Slides were then mounted with Vectashield for nuclei staining. Images were analysed with Nikon confocal microscope and Volocity software (PerkinElmer, Waltham, MA, USA).

**Immunoblotting**

The assay was performed using standard laboratory protocols. Briefly, cells were lysed with RIPA lysis buffer on ice for 30 min and centrifugated at 12,000 rpm for 20 min at 4°C. The protein in the supernatant was collected and quantified using Pierce BCA Protein Assay Kit (Thermo Fisher Scientific, Waltham, MA, USA). A total of 40 μg of proteins were loaded into SDS-PAGE gels, followed by immunoblotting with different primary antibodies. β-actin antibodies were used as the loading control. Signals were detected with ECL reagents (Thermo Fisher Scientific), and the images were captured and analysed using Amersham Imager 600 (GE Healthcare, USA). The relative quantity of each band was analysed with the ImageJ software (NIH, Bethesda, MD, USA).

**Flow cytometry (FCM)**

Flow cytometry was performed using standard laboratory protocols. Briefly, PE/Cy7-conjugated anti-mouse CD45, PE-conjugated anti-mouse CD11b, and PerCP/Cy5.5-conjugated anti-mouse F4/80 antibodies were added directly into the intrahepatic infiltrated cell suspensions for 30 min in the dark at 4°C. After twice washing, cells were resuspended in PBS containing 0.1% BSA and 1 mM EDTA. Data were acquired in LSR-II (BD, CA, USA) and analysed using Flowjo software (Tree Star, OR, USA).

**Quantitative real-time PCR (qRT-PCR)**

Total mRNA from the cells or liver tissues were isolated using TRIzol (Thermo Fisher Scientific) following the manufacturer's protocol. Gene transcriptional levels were determined by qRT-PCR using SYBR Green reagent (Takara, Dalian, China) on an Applied Biosystems 7500 Real-Time PCR system (Life Technologies, Carlsbad, CA, USA) with primers listed in **Table S3**. The primers were synthesized in SinoGenoMax. The cDNA was synthesized using PrimeScript RT Reagents (Takara). The PCR conditions were 3 min at 95°C, followed by 2-step cycles of 5 sec at 95°C and 35 sec at 60°C for total of 40 rounds, and then 5 min at 72°C. Each sample was determined in triplicates. The relative transcriptional levels were determined with GAPDH as control.

**Table S1.** **Primers used for detection of HBV cccDNA, amplification and sequencing**

**of PreS/S regions**

| **Primer** |  | | **Primer Sequence （5’ to 3’）** | **Location** | | |  |
| --- | --- | --- | --- | --- | --- | --- | --- |
| Detection of replication-comment HBV | | | | | | |  |
| HBV-FW | |  | CGTCTGTGCCTTCTCATCTGC | | | 1550-1570 |  |
| HBV-RE | |  | GCACAGCTTGGAGGCTTGAA | | | 1882-1863 |  |
| HBV-probe | |  | TCACCT CTGCCTAATCATCTC | | | 1825–1845 |  |
| Sequencing of PreS/S | | | | |  | | |
| S-FW outside | |  | TTTGCGGGTCACCATATTCTTGG | | | 2815–2837 |  |
| S-RE outside | |  | CGAACCACTGAACAAATGGCACTAG | | | 704–680 |  |
| S-FW inside | |  | CTACAGCATGGGAGGTTGGT | | | 2849–2868 |  |
| S-RE inside | |  | CCAAGAGAAACGGACTGAGG | | | 670–651 |  |

**Table S2.** **Antibodies and ELISA kits**

| **Antibody against** | **Clone** | **RRID** | **Cat. Num** | **Source** | **Application** |
| --- | --- | --- | --- | --- | --- |
| HBsAg | H2F4 | AB_10856214 | bsm-2024M | Bioss | IHC, IF |
| human/mouse CD45 | polyclonal | AB_10853691 | bs-0522R | Bioss | IHC |
| human/mouse IRE1a | 14C10 | AB_823545 | 3294 | Cell Signaling Technology | IB |
| human LASS6(CerS6) | L-18 | AB_2133114 | sc-100554 | Santa Cruz | IB |
| human/mouse/rat CHOP | polyclonal | AB_2292610 | 15204-1-AP | Proteintech | IB |
| human/mouse LASS2(CerS2) | polyclonal | AB_2878677 | 20344-1-AP | Proteintech | IB |
| human/mouse XBP1 | EPR4086 | AB_10861160 | ab109221 | Abcam | IB |
| human/mouse NLRP3 | D4D8T | AB_2722591 | 15101 | Cell Signaling Technology | IB |
| mouse cleaved Caspase-1(p20) | Casper-1 | AB_2490248 | AG-20B-0042 | AdipoGen | IB |
| human/mouse BiP (GRP78) | C50B12 | AB_2119845 | 3177 | Cell Signaling Technology | IB |
| β-actin | AC-40 | AB_262137 | A3853 | Sigma-Aldrich | IB |
| Cy3 Anti-Goat IgG (H+L) | Polyclonal | AB_2340411 | 705-105-003 | Jackson ImmunoResearch | IF |
| human GRP78 | N-20 | AB_631616 | sc-1050 | Santa Cruz | IF |
| Cy3 Anti-Rabbit IgG (H+L) | Polyclonal | AB_2338000 | 111-165-003 | Jackson ImmunoResearch | IF |
| FITC Anti-mouse IgG | Polyclonal | N/A | F0261 | Dako | IF |
| human calnexin | polyclonal | AB_2069033 | 10427-2-AP | Proteintech | IF |
| mouse F4/80-PerCP/Cy5.5 | BM8 | AB_914345 | 45-4801-82 | Thermo Fisher | FC |
| mouse CD45-PE/Cy7 | 30-F11 | AB_469625 | 25-0451-81 | Thermo Fisher | FC |
| mouse CD11b-PE | M1/70 | AB_2734869 | 12-0112-82 | Thermo Fisher | FC |
|  |  |  |  |  |  |
| Mouse IL-1 beta | N/A | N/A | 88-8014 | Thermo Fisher | ELISA |
| Mouse IL-18 | N/A | N/A | BMS618-3 | Thermo Fisher | ELISA |
| Mouse IL-23p19 | N/A | N/A | 88-7230 | Thermo Fisher | ELISA |
| Mito-Tracker Red CMXRos | N/A | N/A | C1035 | Beyotime | IF |
| HBsAg | N/A | N/A | NMPN*: S10910113 | Kehua | ELISA |

IHC: Immunohistochemistry; IF: Immunofluorescence; IB: Immunoblotting; FC: Flow Cytometry; ELISA: Enzyme Linked Immunosorbent Assay; N/A: Not applicable. *NMPN, national medicine permission number.

**Table S3.** **Primers used in qRT-PCR analysis**

| **Genes (protein)** | **Gene ID** | **Primer Sequence（5’ to 3’）** | | **Location** | **F/R** | **Size** | |
| --- | --- | --- | --- | --- | --- | --- | --- |
| hu*GRP78* (GRP78) | 3309 | CATCACGCCGTCCTATGTCG | | 397-416 | F | 104bp | |
|  |  | CGTCAAAGACCGTGTTCTCG | | 500-481 | R |  |  |
| hu*ERN1* (IRE1a) | 2081 | AGAGGACAGGCTCAATCAAATGG | | 255-277 | F | 297bp | |
|  |  | TATCTGCAAAGGCCGATGACAAA | | 551-529 | R |  |  |
| hu*DDIT3*  *(*CHOP) | 1649 | GGAAACAGAGTGGTCATTCCC | | 480-500 | F | 116bp | |
|  |  | CTGCTTGAGCCGTTCATTCTC | | 595-575 | R |  |  |
| huXBP1  (XBP1) | 7494 | CCCTCCAGAACATCTCCCCAT | | 564-584 | F | 101bp | |
|  |  | ACATGACTGGGTCCAAGTTGT | | 664-644 | R |  |  |
| hu*ATF6*  (ATF6) | 22926 | TCCTCGGTCAGTGGACTCTTA | | 312-332 | F | 235bp | |
|  |  | CTTGGGCTGAATTGAAGGTTTTG | | 546-524 | R |  |  |
| hu*EIF2AK3* (PERK) | 9451 | ACGATGAGACAGAGTTGCGAC | | 278-298 | F | 80bp | |
|  |  | ATCCAAGGCAGCAATTCTCCC | | 357-337 | R |  |  |
| hu*CERS1* (CERS1) | 10715 | ACGCTACGCTATACATGGACAC | | 499-520 | F | 87bp | |
|  |  | AGGAGACGATGAGGATGAG | | 585-567 | R |  |  |
| hu*CERS2* (CERS2) | 29956 | CCGATTACCTGCTGGAGTCAG | | 983-1003 | F | 83bp | |
|  |  | GGCGAAGACGATGAAGATGTTG | | 1065-1044 | R |  |  |
| hu*CERS3* (CERS3) | 204219 | ACATTCCACAAGGCAACCATTG | | 493-514 | F | 109bp | |
|  |  | CTCTTGATTCCGCCGACTCC | | 601-582 | R |  |  |
| hu*CERS4* (CERS4) | 79603 | CTTCGTGGCGGTCATCCTG | | 922-940 | F | 78bp | |
|  |  | TGTAACAGCAGCACCAGAGAG | | 999-979 | R |  |  |
| hu*CERS5* (CERS5) | 91012 | GTTTCGCCATCGGAGGAATC | | 648-667 | F | 152bp | |
|  |  | GCCAGCACTGTCGGATGTC | | 799-781 | R |  |  |
| hu*CERS6* (CERS6) | 253782 | GGGATCTTAGCCTGGTTCTGG | | 184-204 | F | 83bp | |
|  |  | GCCTCCTCCGTGTTCTTCAG | | 266-247 | R |  |  |
| hu*DEGS1* (DEGS1) | 8560 | GAGATCCTGGCAAAGTATCCAGA | | 170-192 | F | 157bp | |
|  |  | CAAACGCATAGGCCCCAAA | | 326-308 | R |  |  |
| hu*GAPDH* (GAPDH) | 2597 | GGAGCGAGATCCCTCCAAAAT | | 108-128 | F | 197bp | |
|  |  | GGCTGTTGTCATACTTCTCATGG | | 304-282 | R |  |  |
| m*Cers1*  (Cers1) | 93898 | GCCACCACACACATCTTTCGG  GGAGCAGGTAAGCGCAGTAG | | 314-334  446-427 | F  R | 133bp | |
| m*Cers2*  (Cers2) | 76893 | TATGACTACTTCTGGTGGGAACG | | 16-38 | F | 147bp | |
|  |  | GTATCGAATGACGAGAAAGAGCA | | 162-140 | R |  |  |
| m*Cers3*  (Cers3) | 545975 | CCTGGCTGCTATTAGTCTGATG  CTGCTTCCATCCAGCATAGG | | 983-1004  1121-1102 | F  R | 139bp | |
| m*Cers4*  (Cers4) | 67260 | CTGTGGTACTGTTGTTGCATGAC  GCGCGTGTAGAAGAAGACTAA | | 885-907  1015-995 | F  R | 131bp | |
| m*Cers5*  (Cers5) | 71949 | TGCTGTTTGAGCGATTTATTGC  GGTTCCACCTTATTGACAGGAC | | 321-342  399-378 | F  R | 79bp | |
| m*Cers6*  (Cers6) | 241447 | GTTCTGGAACGAGCGGTTTTG | | 21-41 | F | 181bp | |
|  |  | GAGGGCTATGGCACATGGTT | | 201-182 | R |  |  |
| m*Degs1* | 13244 | GAATGGGTCTACACGGACCAG | | 31-51 | F | 225bp | |
| (Degs1) |  | AGTCATGGAGTGGTTAAGGCA | | 255-235 | R |  |  |
| m*Gapdh* (Gapdh) | 14433 | AGGTCGGTGTGAACGGATTTG | | 8-28 | F | 123 bp | |
|  |  | TGTAGACCATGTAGTTGAGGTCA | | 130-108 | R |  |  |
| **Table S4.** **Histology-confirmed 1823 HCC patients with different serologically viral markers*** | | | | | | |  |
| **HBV and/or HCV serological markers** | | | **Number (%)** | | | |  |
| Infection with HBV alone | | | 1567 (86.0%) | | | |  |
| HBsAg-pos&anti-HBc-pos&anti-HCV-neg | | | 1331 (73.0%) | | | |  |
| HBsAg-neg&anti-HBc-pos&anti-HCV-neg | | | 236 (12.9%) | | | |  |
| Infection with HCV alone | | |  | | | |  |
| HBsAg-neg& anti-HBc-neg& anti-HCV-pos | | | 46 (2.5%) | | | |  |
| Co-infection with HBV and HCV | | | 122 (6.7%) | | | |  |
| HBsAg-pos & anti-HBc-pos& anti-HCV-pos | | | 27 (1.5%) | | | |  |
| HBsAg-neg& anti-HBc-pos & anti-HCV-pos | | | 95 (5.2%) | | | |  |
| No infection with HBV or HCV | | |  | | | |  |
| HBsAg-neg& anti-HBc-neg & anti-HCV-neg | | | 88 (4.8%) | | | |  |

HBV, hepatitis B virus; HCV, hepatitis C virus; HCC, hepatocellular carcinoma; HBsAg, hepatitis B surface antigen; anti-HBc, antibodies against hepatitis B virus core antigen; anti-HCV, antibodies against hepatitis C virus.

*Derived from Wang M., et al. in International Journal of Infectious Diseases. 2017. 65: p. 15-21

Title: Contribution of hepatitis B virus and hepatitis C virus to liver cancer in China north areas: Experience of the Chinese National Cancer Center.

**Table S5.** **The amounts of specified species of ceramide determined by HPLC-MS/MS in HepG2 cells that were differently treated for 24 h after HBV plasmid transfection**

| Species | Amounts in empty-vector-transfected HepG2 (pmol/mg protein) | **Folds relative to empty-vector-transfected HepG2** | | | | | | | | |
| --- | --- | --- | --- | --- | --- | --- | --- | --- | --- | --- |
|  |  | Solvent treatment (fatty acid-free BSA) | | | 250 μM palmitic acid treatment | | | treatment with 250 μM palmitic acid & 5 μM myriocin | | |
|  |  | Ref-HBV | mtPreS1 | mtPreS2 | Ref-HBV | mtPreS1 | mtPreS2 | Ref-HBV | mtPreS1 | mtPreS2 |
| total | 876.58±3.24 | 1.66±0.03 | 2.42±0.10 | 2.61±0.09 | 3.14±0.05 | 4.42±0.04 | 6.14±0.09 | 1.44±0.02 | 2.16±0.05 | 2.17±0.02 |
| C14:0 | 4.51±0.01 | 1.47±0.02 | 1.66±0.07 | 1.95±0.07 | 3.34±0.05 | 5.83±0.04 | 5.56±0.06 | 2.17±0.02 | 3.38±0.07 | 4.08±0.02 |
| C16:0 | 15.23±0.05 | 2.95±0.04 | 4.09±0.17 | 4.32±0.15 | 5.57±0.08 | 8.78±0.05 | 8.90±0.09 | 2.81±0.02 | 4.46±0.09 | 4.76±0.03 |
| C18:0 | 1.94±0.01 | 1.19±0.01 | 1.91±0.08 | 1.79±0.06 | 2.16±0.03 | 3.52±0.02 | 4.15±0.04 | 2.42±0.02 | 4.09±0.08 | 5.25±0.03 |
| C18:1 | 1.56±0.01 | 1.91±0.02 | 2.85±0.12 | 3.45±0.12 | 3.24±0.05 | 5.16±0.03 | 6.59±0.07 | 1.35±0.01 | 2.08±0.04 | 2.39±0.02 |
| C20:0 | 18.37±0.05 | 1.31±0.02 | 2.44±0.10 | 2.13±0.07 | 2.87±0.04 | 4.58±0.03 | 5.64±0.06 | 1.85±0.02 | 3.46±0.07 | 3.61±0.02 |
| C22:0 | 116.59±0.49 | 1.82±0.02 | 3.11±0.13 | 3.21±0.11 | 4.55±0.07 | 6.58±0.04 | 7.64±0.47 | 1.29±0.01 | 2.54±0.05 | 2.69±0.02 |
| C24:0 | 226.55±1.28 | 1.95±0.02 | 2.29±0.10 | 2.65±0.09 | 3.26±0.22 | 4.11±0.02 | 6.31±0.06 | 1.24±0.01 | 1.31±0.03 | 1.27±0.01 |
| C24:1 | 463.11±1.48 | 1.51±0.02 | 2.36±0.10 | 2.52±0.09 | 3.33±0.23 | 5.25±0.03 | 7.10±0.07 | 1.53±0.01 | 2.47±0.05 | 2.49±0.02 |
| C26:0 | 5.57±0.03 | 1.27±0.02 | 1.40±0.06 | 2.01±0.07 | 1.88±0.03 | 2.62±0.02 | 4.79±0.05 | 1.03±0.01 | 1.12±0.02 | 1.59±0.01 |
| C26:1 | 31.79±0.12 | 1.28±0.02 | 1.45±0.06 | 1.57±0.06 | 3.03±0.04 | 3.89±0.02 | 5.62±0.06 | 1.34±0.01 | 1.53±0.03 | 1.07±0.01 |

Shown (mean ± SD) is one representative of three independent experiments, that were quantified in triplicates.


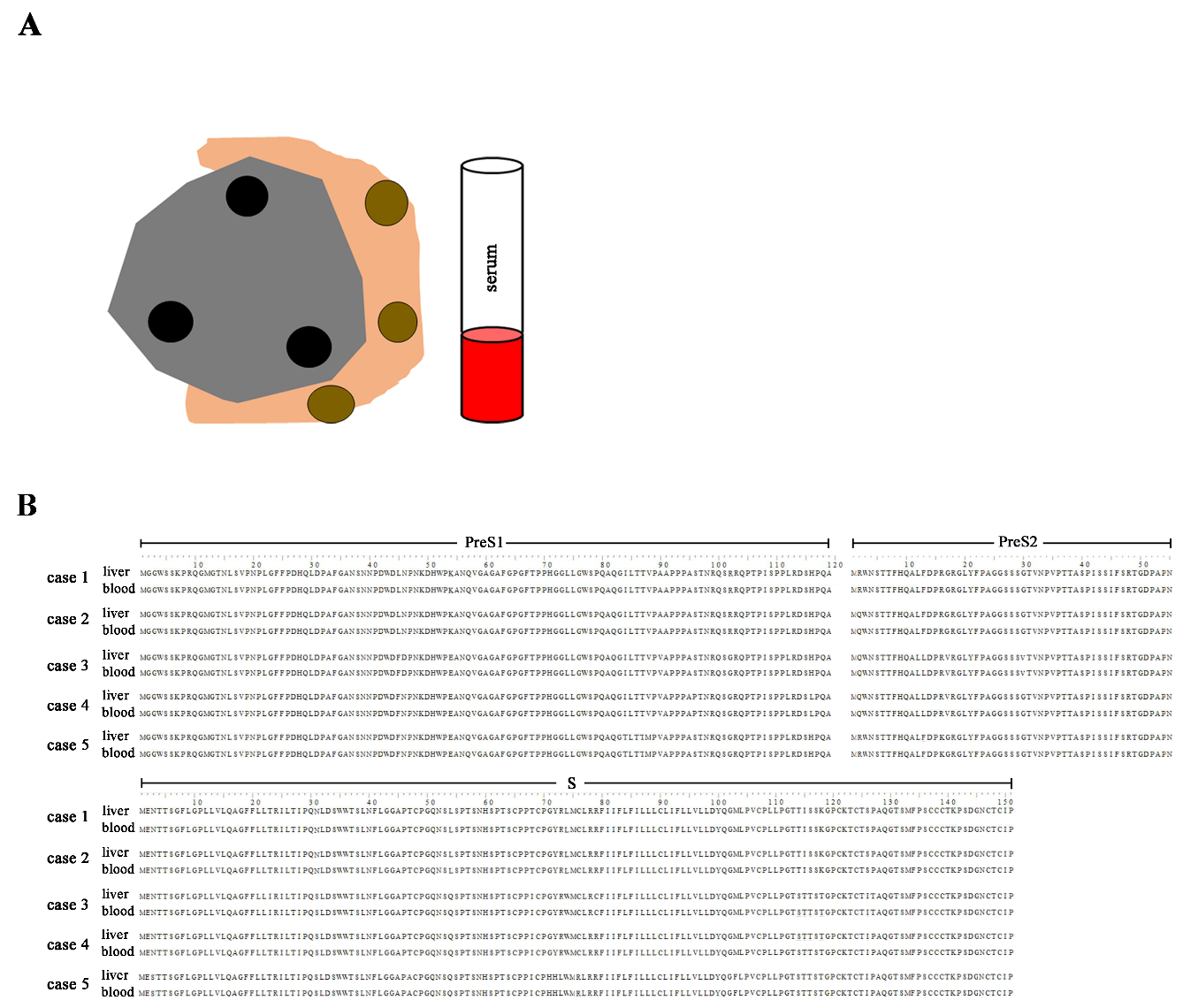


**Figure S1.** **Analysis of HBV PreS/S regions in 35 HCC patients with serological markers of HBsAg (-) &anti-HBc (+) &anti-HCV (-)**

**A)** Diagram for tissue sampling, each circle represents one piece from each HCC case. HBV was also analysed in the matched serum. **B)** Sequences of PreS/S regions isolated from liver tissues and from bloods in five representative HBsAg-seronegative HCC patients. All the sequences were deposited into GenBank with accession numbers of MW422170: MW422204.


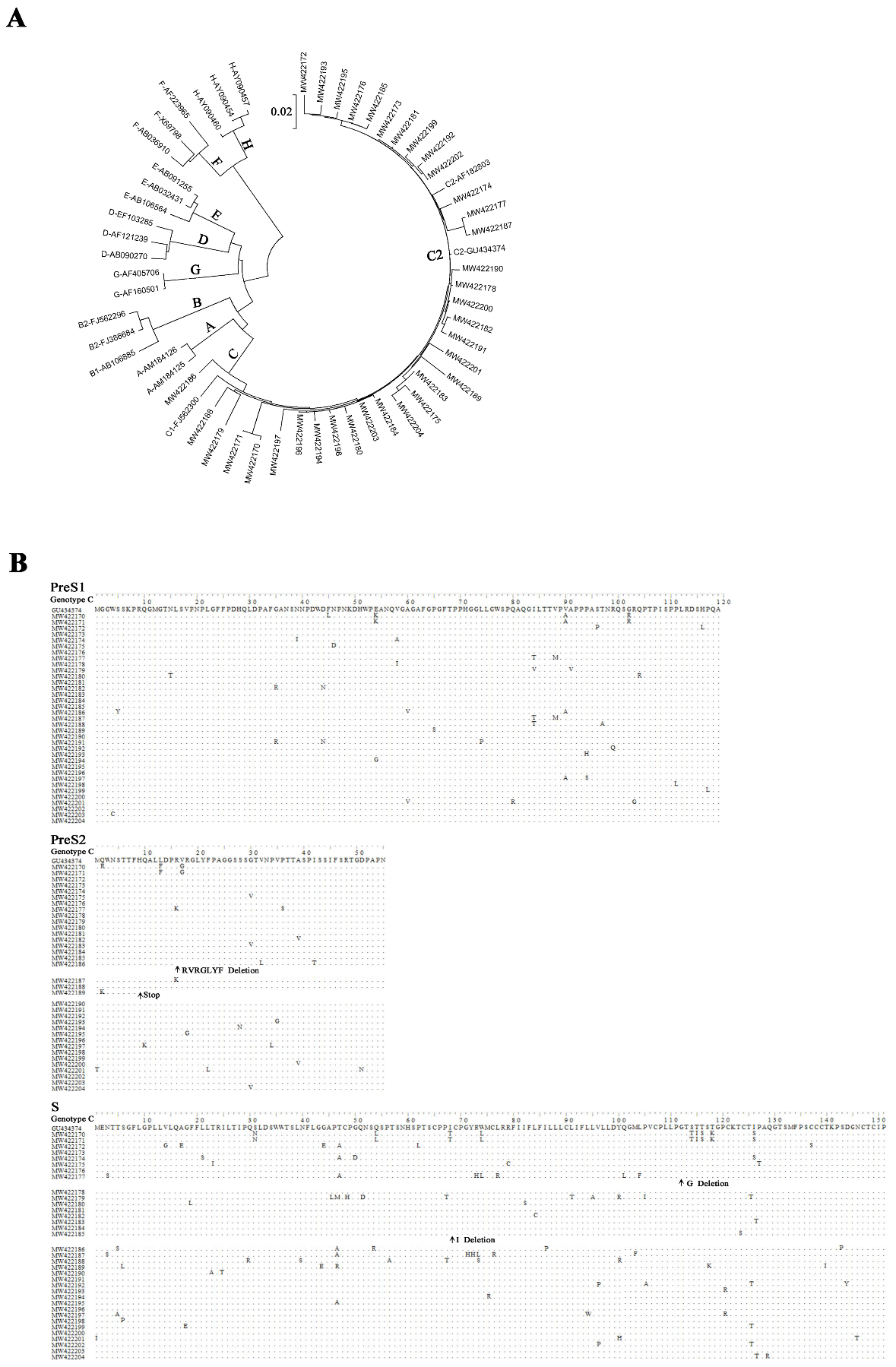


**Figure S2.** **Aliment of 35 HBV isolates from the HBsAg-seronegative HCC patients**

**A)** Phylogenetic tree of HBV isolates based on the sequenced PreS/S regions analysed with MEGA5.05 software. **B)** Alignment of deduced HBV large envelop proteins from the 35 isolates, their GenBank accession numbers are MW422170: MW422204. The reference HBV is an isolate (genotype C), GenBank accession number GU434374, that was obtained from one HBsAg-seropositive HCC patient.


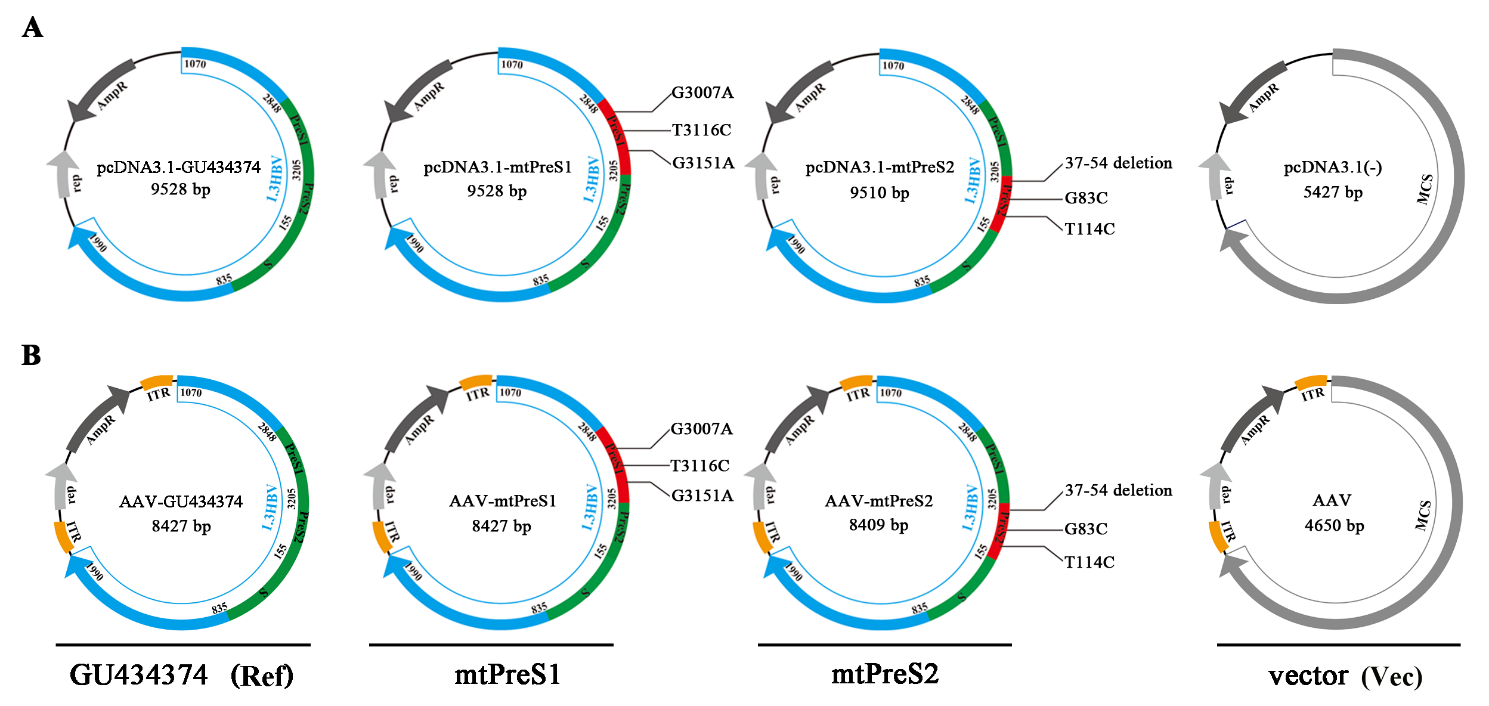


**Figure S3.** **Profile of the HBV replicating plasmids**

**A)** The pcDNA3.1(-) vector was used to construct three different HBV plasmids for cell transfection. Each HBV plasmid contains 1.3×HBV genome, and mtPreS1 differs only in PreS1, mtPreS2 differs only in PreS2 from reference HBV (GenBank accession number: GU434374) which was isolated from one HBsAg-seropositive HCC patient. **B)** The AAV-MCS vector was used to construct three different HBV plasmids for mice liver transfection. Each HBV plasmid contains 1.3×HBV genome.


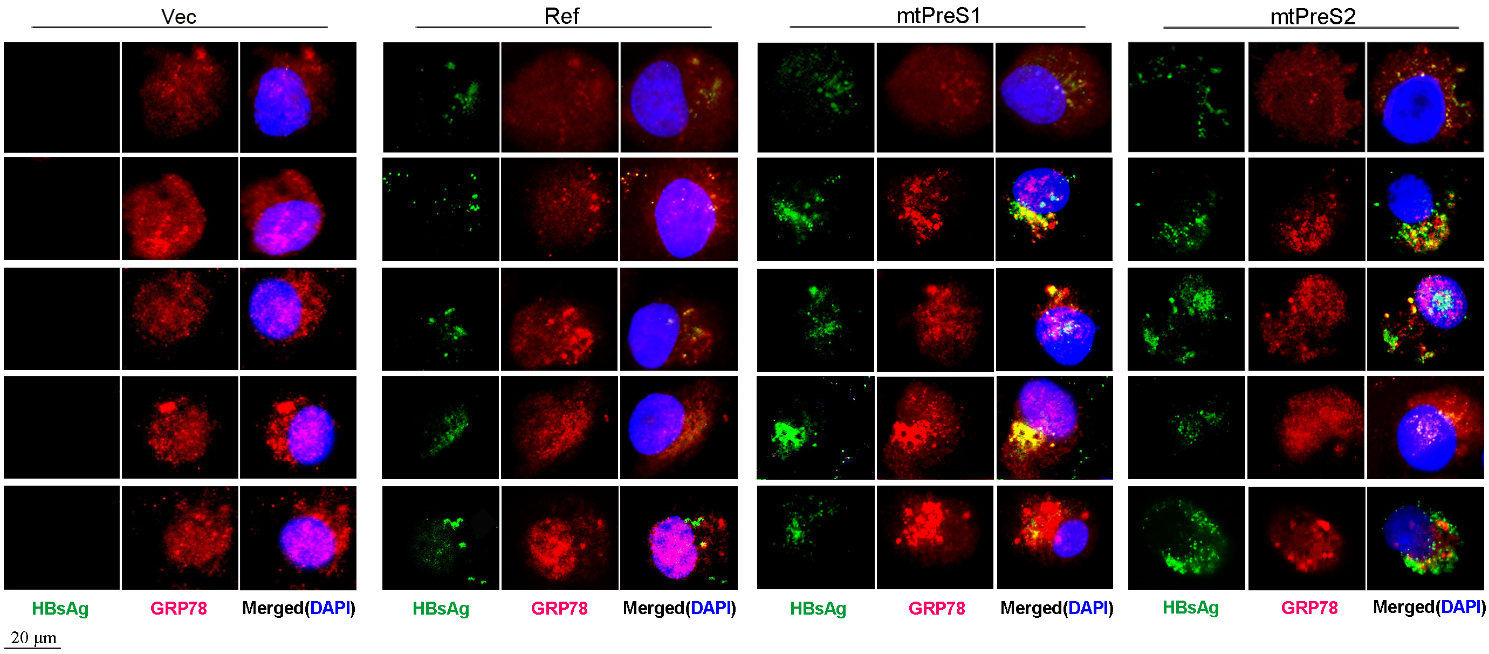


**Figure S4.** **The staining of envelop proteins and hepatocyte ER in five independent experiments**

The maps of plasmids were depicted in Fig. S3A. HepG2 cells (5×10^5^/well in a 6-well plate) were transfected with 2.5 μg of indicated HBV plasmids respectively and cultured for 48 h, that were Ref-HBV (Ref), mtPreS1, mtPreS2. Empty-vector, pcDNA3.1(-), was used as transfection control (Vec). The transfected cells were spined onto a slide and stained with goat anti-human GRP78 (Santa Cruz), and mouse anti-HBsAg monoclonal antibodies at 4 °C overnight. FITC-labeled anti-mouse IgG and Cy3-labeled anti-goat IgG were then added for 1 h at room temperature. The stained slides were mounted with Vectashield for nuclei staining. The images were analysed with Leica microscope and LAS software (Wentzler, Germany). Representative microscopy images of five independent experiments (from top to bottom) show the staining of envelop proteins (HBsAg, green) and ER (GRP78, red). Nuclei were stained with DAPI (blue).


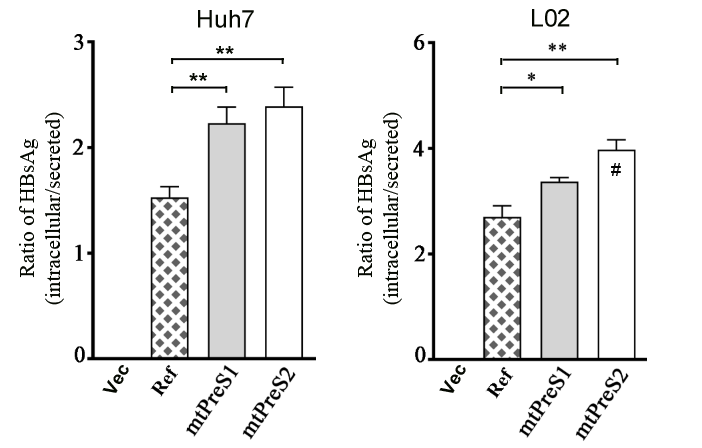


**Figure S5.** **HBsAg secretion from hepatocytes after transfection with different HBV plasmids**

Equal numbers (5×10^5^/well in a 6-well plate) of Huh7 cells or L02 cells were transfected with 2.5 μg of indicated plasmids respectively and cultured for 48 h, that were Ref-HBV (Ref), mtPreS1, mtPreS2. Empty-vector, pcDNA3.1(-), was used as transfection control (Vec). The supernatant was collected, cells were washed twice with PBS and dissolved in RIPA lysis buffer. The envelop protein (HBsAg) in supernatant (secreted) and in total cell lysates (intracellular) were quantified using a commercialized HBsAg ELISA kit. Bar graphs (mean ± SEM) show the ratio of intracellular to secreted HBsAg after normalizing to total amounts of cellular proteins from three independent experiments. Each group was triplicated in one independent experiment. Normal distribution of variables was examined by Shapiro-Wilk normality test. The differences of HBsAg secretion from hepatocytes transfected with different plasmids were compared with one-way ANOVA analysis. **P*< 0.05, ***P*< 0.01 between Ref-HBV and mtPreS1 or mtPreS2; ^#^*P*< 0.05 between mtPreS1 and mtPreS2.


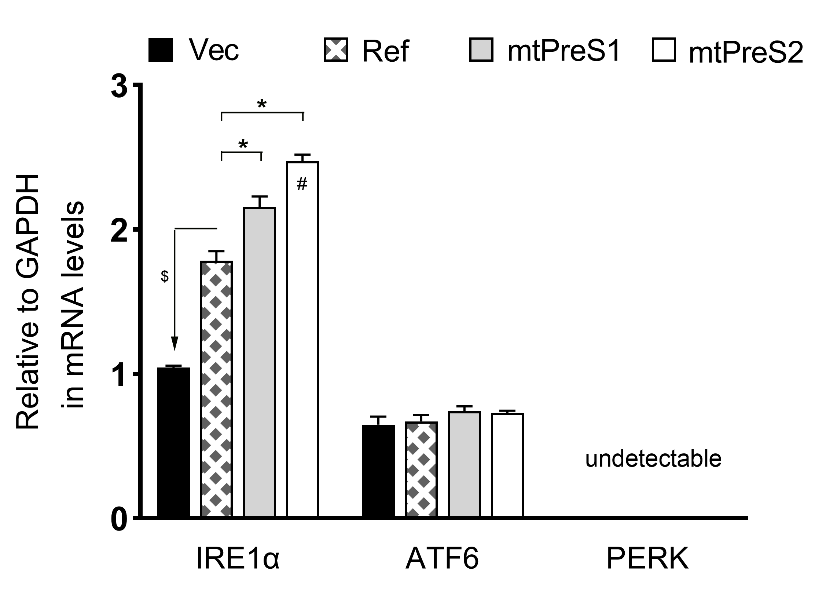


**Figure S6.** **Transcription levels of IRE1α, ATF6, and PERK in HepG2 after transfection with different HBV plasmids**

HepG2 cells were transfected with Ref-HBV (Ref), or mtPreS1, or mtPreS2, or empty-vector (Vec), respectively, and cultured for 48 h without any stimulation. Bar graph shows transcriptional levels (mean ± SEM) of *IRE1α*, *ATF6,* and *PERK* from three independent experiments determined by qRT-PCR. Normal distribution of variables was examined by Shapiro-Wilk normality test. The differences of gene expression of hepatocytes transfected with different plasmids were compared with one-way ANOVA analysis. ^$^*P*< 0.05 between Ref-HBV and empty-vector; **P*< 0.05 between Ref-HBV and mtPreS1 or mtPreS2; ^#^*P*< 0.05 between mtPreS1 and mtPreS2.


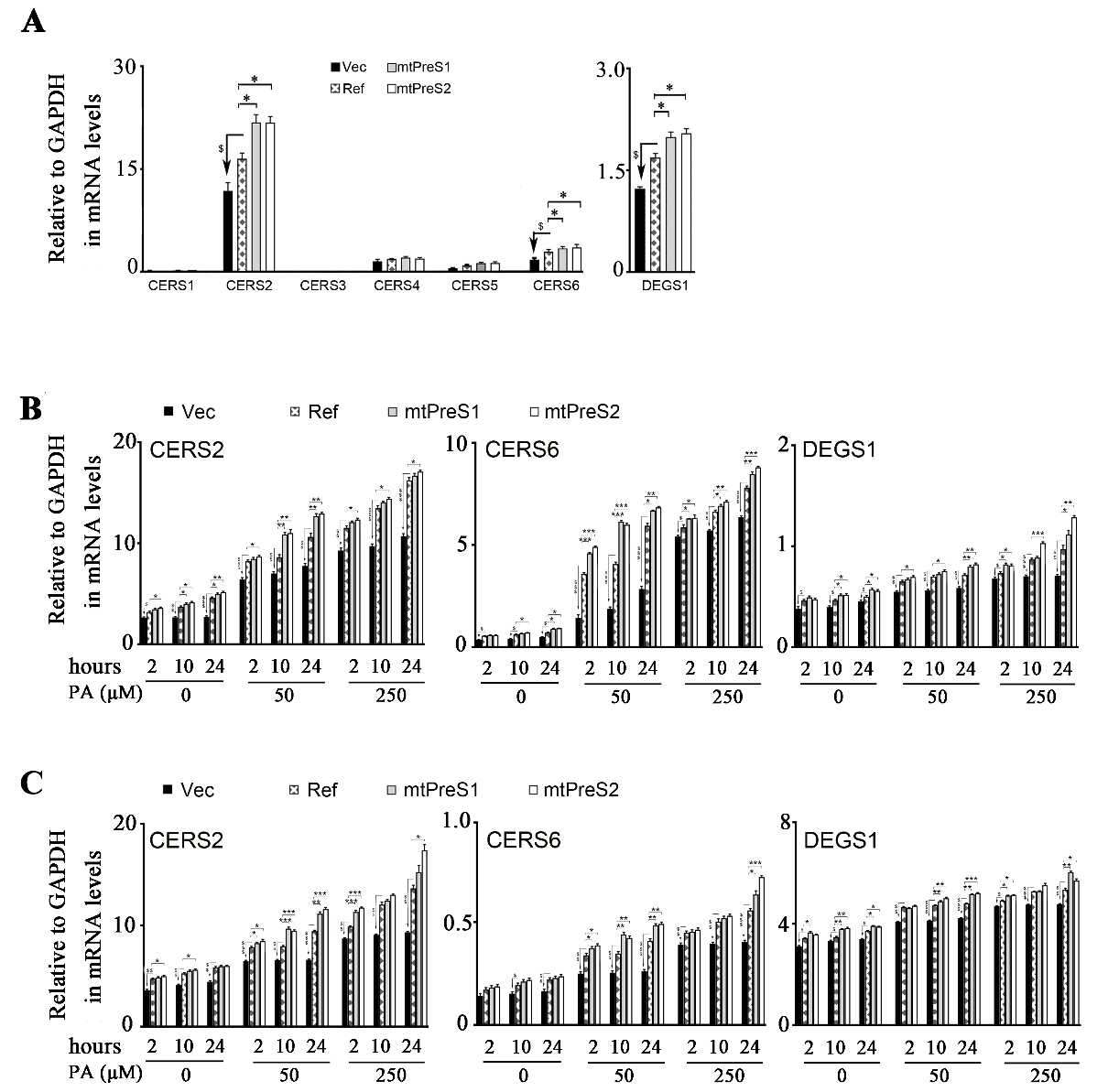


**Figure S7.** **Transcriptional levels of *CERS1-6* and *DEGS1***

**A)** HepG2 cells were transfected with Ref-HBV (Ref), or mtPreS1, or mtPreS2, or empty-vector (Vec), respectively, and continued to culture for 48 h without any stimulation. Bar graph (mean ± SEM) shows transcriptional levels of *CERS1-6* and *DEGS1* from three independent experiments. **B)** Huh7 cells, **C)** L02 cells were transfected with different HBV plasmids as did in Figure 3C. The cells, 48 h later, were replaced with medium that contained 0, 50, or 250 μM of PA for indicated time for analysis. Shown is one representative of three independent experiments. The 0-μM represents the medium containing 0.25% of FF-BSA and NaOH. Normal distribution of variables was examined by Shapiro-Wilk normality test. One-way ANOVA analysis was used to compare the difference. ^$^*P*< 0.05, ^$$^*P*< 0.01, ^$$$^*P*< 0.001 between Ref-HBV and empty-vector; **P*< 0.05, ***P*< 0.01, ****P*< 0.001 between Ref-HBV and mtPreS1 or mtPreS2. No statistical difference was observed between mtPreS1 and mtPreS2 in Figure S7A. The difference between mtPreS1 and mtPreS2 was not shown in Figure S7B & S7C.


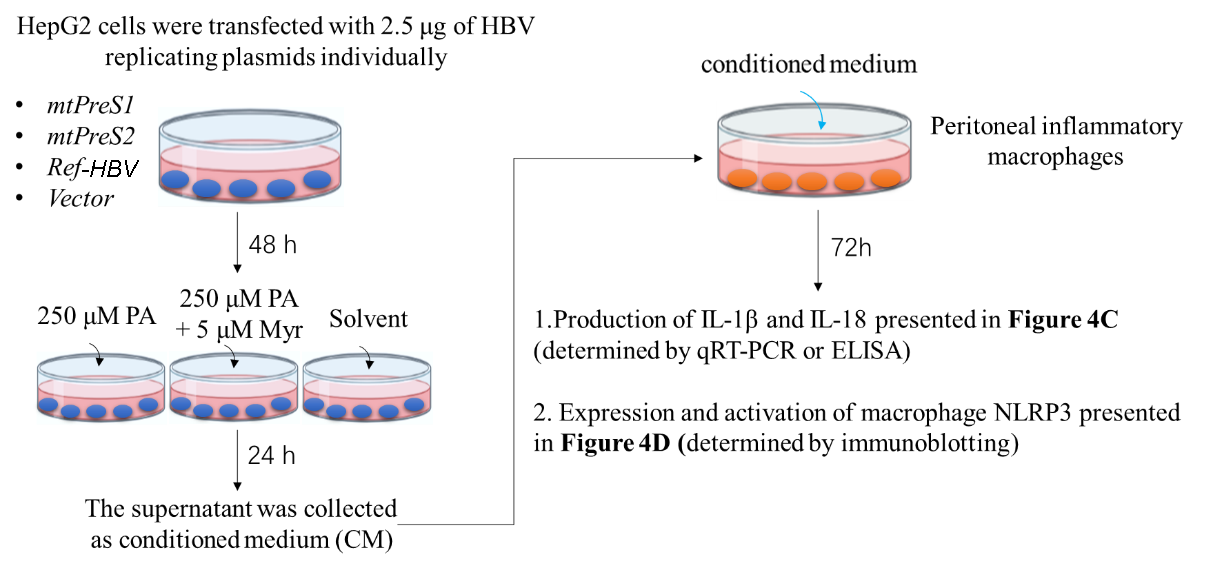


**Figure S8.** **Schematic diagram of culture system to examine the effects of ceramides from hepatocytes that were transfected with different HBV plasmids on inflammatory macrophages**


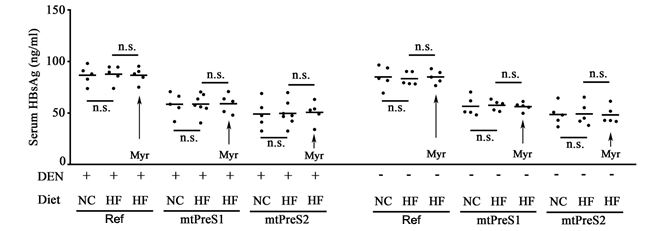


**Figure S9.** **Serum levels of HBsAg in mice that were injected with different HBV plasmids**

The male C57BL/6J mice received different HBV plasmids, that are depicted in Fig. S3B, by intravenous hydrodynamic injection via tail vein. Shown (mean ± SEM) are the serum levels of HBsAg detected in mice at the 8-weeks old, 2 weeks after plasmid injection and before different diet types were given. Each dot indicates one mouse. Normal distribution of variables was examined by Shapiro-Wilk normality test. The differences of serum HBsAg in the same plasmid-injected mice were compared with one-way ANOVA analysis. n.s. indicates no statistical difference. NC, normal chow; HF, high-fat diet; Myr, myriocin.


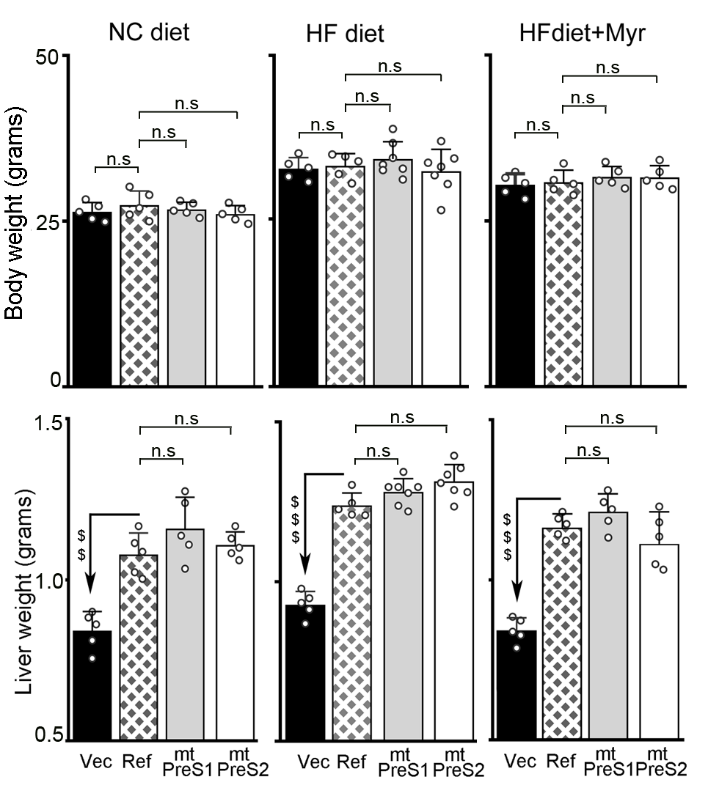


**Figure S10.** **Body weight and liver weight of the mice fed different diet types**

Bar graphs (mean ± SEM) show the body weights (upper panel) and liver weights (low panel) of the different plasmid-injected mice fed different diet types. Empty-vector, AAV-MCS, was used as transfection control (Vec). Each dot indicates one mouse. n=5-7. Normal distribution of variables was examined by Shapiro-Wilk normality test. The differences of differently treated mice were compared by one-way ANOVA analysis. n.s. no statistical difference; ^$$^*P*< 0.01, ^$$$^*P*< 0.001 between Ref-HBV and empty-vector. No statistical difference was observed between Ref-HBV and mtPreS1 or mtPreS2, or between mtPreS1 and mtPreS2. NC, normal chow; HF, high-fat diet; Myr, myriocin.


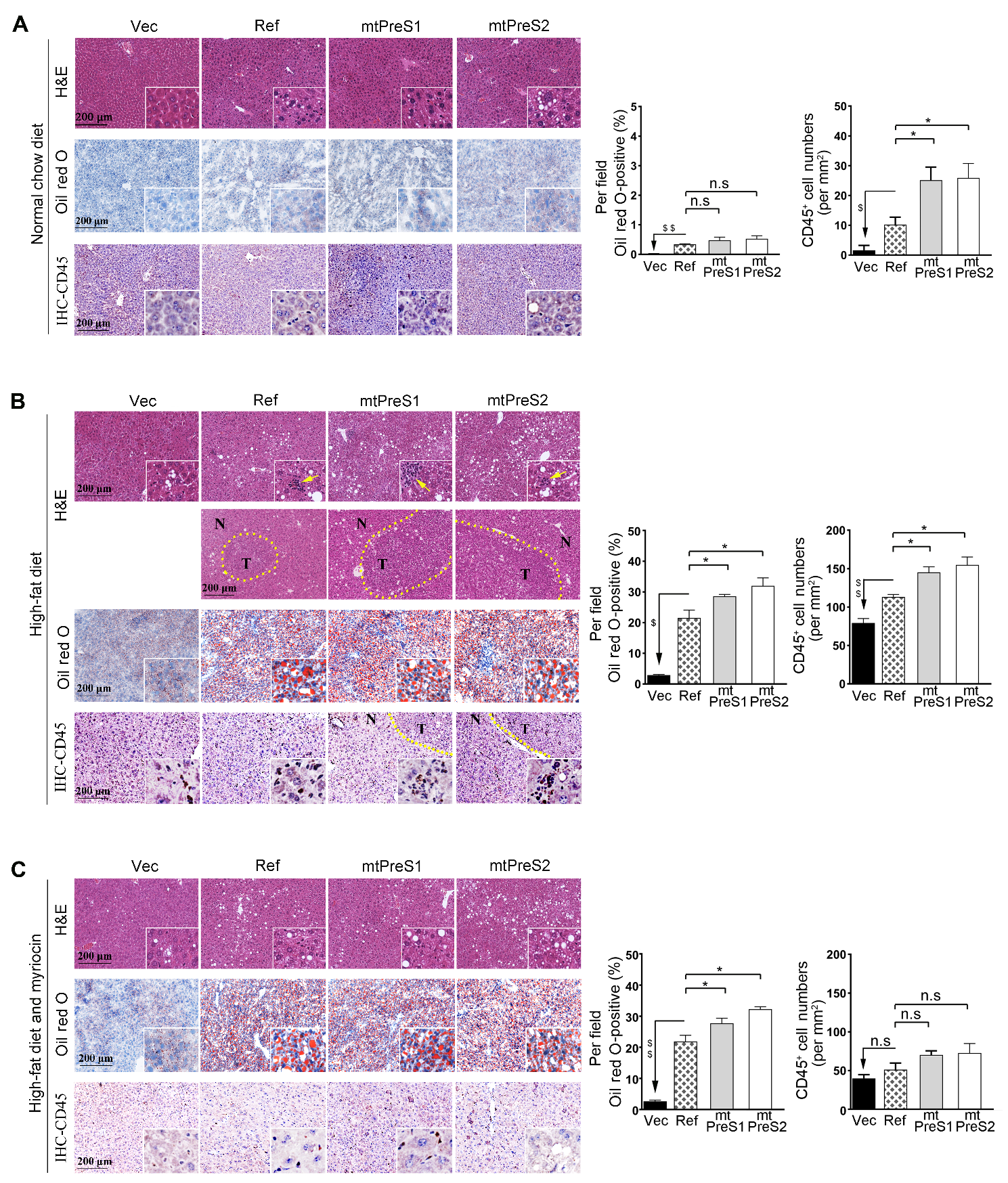


**Figure S11.** **Representative histological images of the DEN-treated mice livers with different diet types**

The mice were sacrificed at the 14-weeks old. For histology analysis, three pictures of each liver sections were taken. Shown are one of the representative images of the mice liver with **A)** normal chow diet, **B)** high-fat diet only, **C)** high-fat diet with myriocin treatment.

H&E staining of tumour-free section (upper panel), the infiltrated inflammatory cells are pointed with arrows in yellow. The liver tumour nodules in HBV-transfected mice fed the HF diet (middle panel in B) are showed and marked with circle in yellow. T: tumour, N: non-tumour. Oil red O staining (middle panel) to detect the hepatic steatosis. Immunohistochemistry staining of CD45 (low panel) to examine the liver inflammation. Bar graphs (mean ± SEM) show the percentage (%) of the areas with positive for Oil red O staining (left panel) and the density of CD45-positive cells (right panel) of the section that were analysed with the ImageJ software (NIH, USA) and Image-Pro Plus 6.0 (Media Cybemetics, USA), respectively. Empty-vector, AAV-MCS, was used as transfection control (Vec). Normal distribution of variables was examined by Shapiro-Wilk normality test. The differences of differently treated mice were compared by one-way ANOVA analysis. n.s. no statistical difference; ^$^*P*< 0.05, ^$$^*P*< 0.01 between Ref-HBV and empty-vector; **P*< 0.05 between Ref-HBV and mtPreS1or mtPreS2; No statistical difference was observed between mtPreS1 and mtPreS2. NC, normal chow; HF, high-fat diet; Myr, myriocin.

When the mice were sacrificed at the 14-weeks old, their liver were divided into five parts. Each part from different mice was the same in anatomy. One part was into neutral formalin for H&E staining and CD45 immunohistochemistry staining (also in Figure 5D), one was frozen-sectioned for Oil red O staining (also in Figure 5D), the other three parts were used respectively for analysis of related gene expression and ceramide generation (Figure 5E), the preparation of liver intercellular fluid (Figure 6C) and the analysis of intrahepatic infiltrated cells (Figure 6B and 6D) of main body.


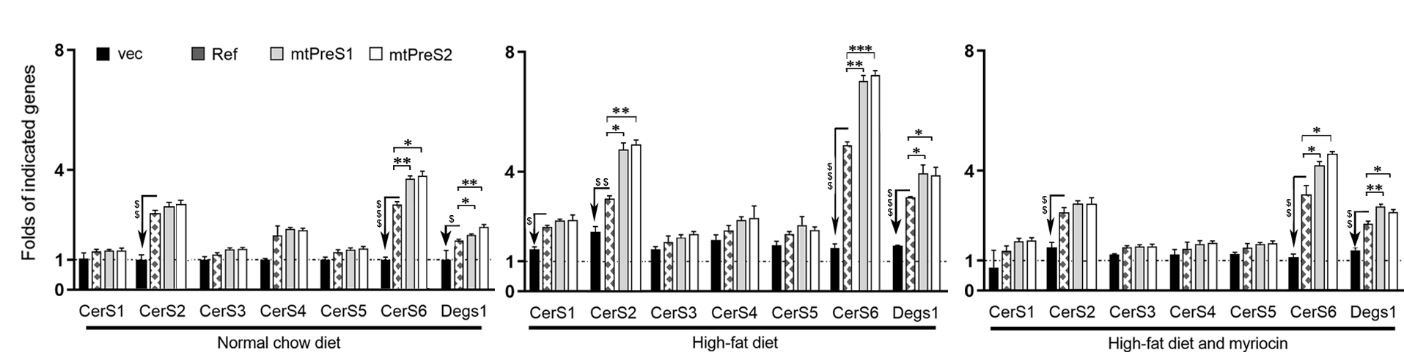


**Figure S12.** **Transcriptional levels of *CerS1-6* and *Degs1* genes in mice livers**

From each group, tumour-free liver tissues from 3 mice were sampled. Transcriptional levels of *CerS1-6* and *Degs1* were determined by qRT-PCR. Shown are changed fold (mean ± SEM) relative to the mice without any transfection and fed the NC diet. Normal distribution of variables was examined by Shapiro-Wilk normality test. One-way ANOVA analysis was used to compare the differences of differently treated mice. ^$^*P*< 0.05, ^$$^*P*< 0.01, ^$$$^*P*< 0.001 between Ref-HBV and empty-vector; **P*< 0.05, ***P*< 0.01, ****P*< 0.001 between Ref-HBV and mtPreS1 or mtPreS2. No statistical difference was observed between mtPreS1 and mtPreS2.


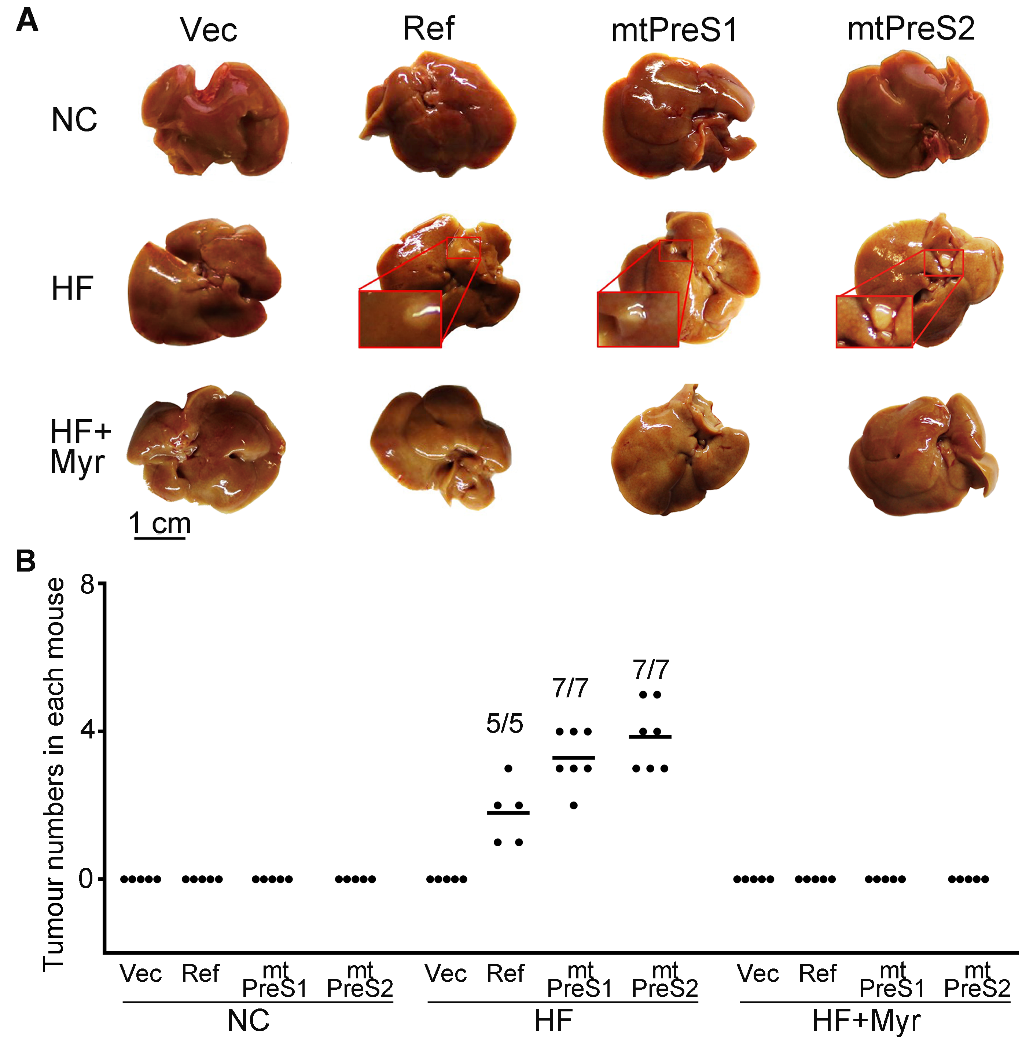


**Figure S13.** **Liver macroscopic appearance and tumour numbers in the DEN-treated mice with different diet types**

The mice were treated as shown in Figure 5A. The C57BL/6 mice received DEN at their 2-weeks old. The same HBV plasmid-injected mice were allocated into two subgroups based on serum HBsAg levels. Beginning at their 8-weeks old, the mice were fed the NC diet or HF diet respectively for five weeks. **A)** Representative images of macroscopic appearance of the liver from DEN injected mice sacrificed at their 14-weeks old. **B)** Tumor numbers in the scarified mice (14-weeks old) that received DEN at their 2-weeks old with different diets from 8 weeks old. Each dot indicates one mouse. The numbers labeled on the top indicate the mice numbers developed liver tumor/total numbers. n=5-7. NC, normal chow; HF, high-fat diet; Myr, myriocin.


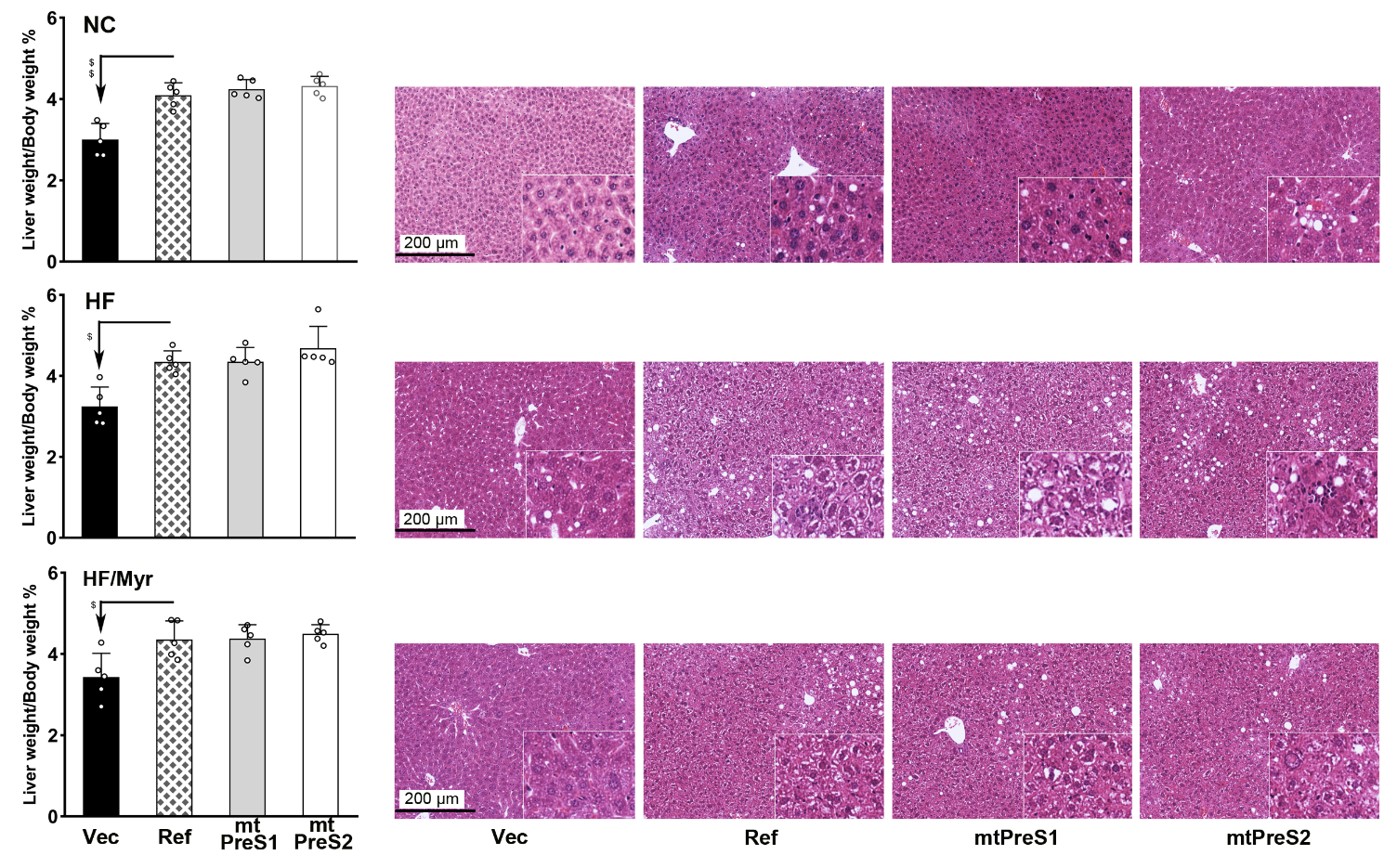


**Figure S14.** **Liver weight and H&E staining of mice livers without DEN injection**

Bar graphs (mean ± SEM) show liver weight to body weight ratios of the mice fed different diets sacrificed at their 14-weeks old that did not receive carcinogen DEN. Images show the representative histology of the mice liver in each group. Three liver sections were prepared from each group. Each liver section taken three pictures. NC, normal chow; HF, high-fat diet; Myr, myriocin. n=5. Normal distribution of variables was examined by Shapiro-Wilk normality test. One-way ANOVA analysis was used to compared the differences of differently treated mice. ^$^*P*< 0.05, ^$$^*P*< 0.01 between Ref-HBV and empty-vector. No statistical difference was observed between Ref-HBV and mtPreS1 or mtPreS2, between mtPreS1 and mtPreS2.


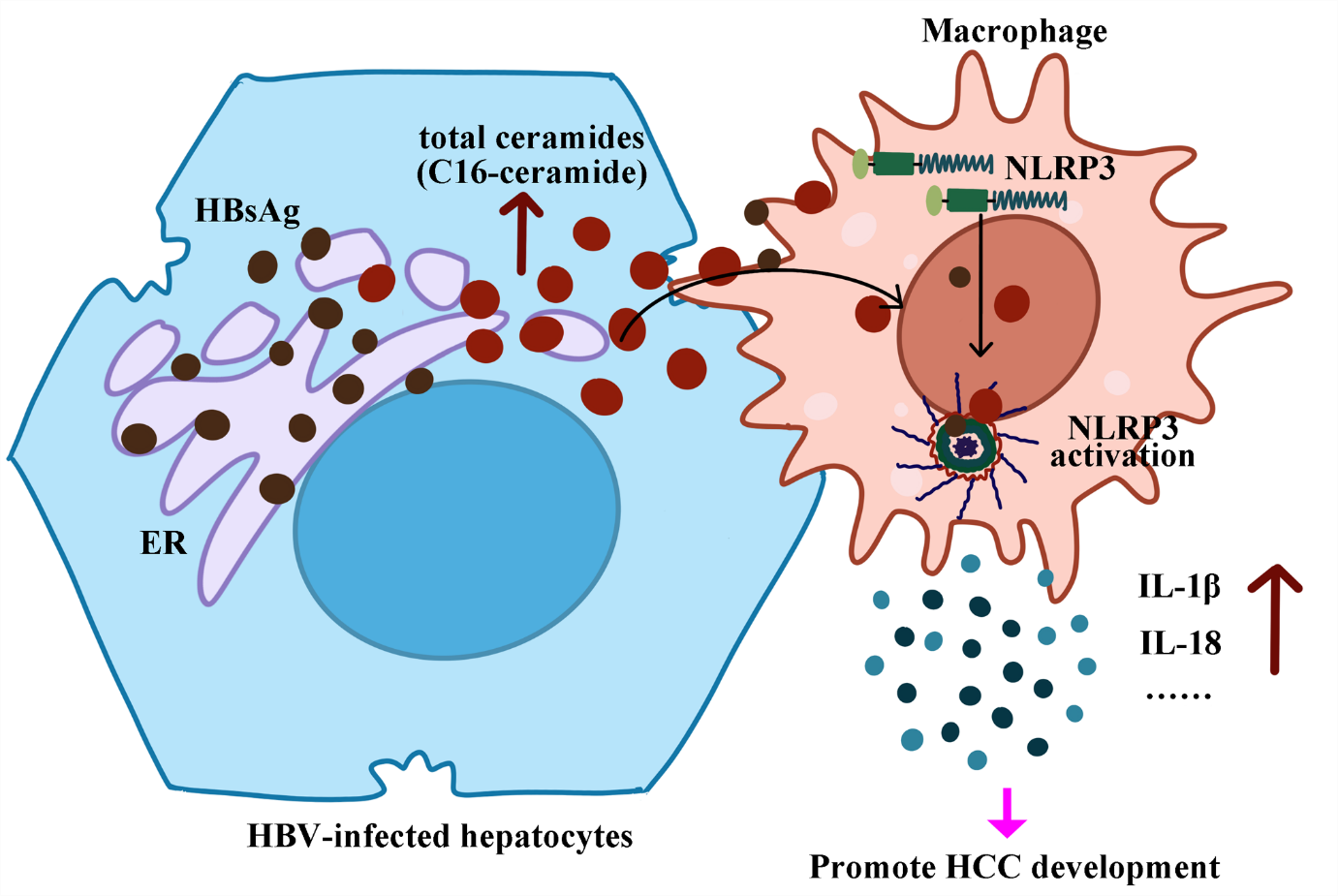


**Figure S15.** **Graphic abstract**
